# Relaxed specificity of BcpB transporters mediates interactions between *Burkholderia cepacia* complex contact-dependent growth inhibition systems

**DOI:** 10.1128/msphere.00303-23

**Published:** 2023-07-27

**Authors:** Zaria K. Elery, Tanya Myers-Morales, Erica D. Phillips, Erin C. Garcia

**Affiliations:** 1 University of Kentucky College of Medicine, Lexington, Kentucky, USA; The University of Iowa, Iowa City, Iowa, USA

**Keywords:** *Burkholderia dolosa*, *Burkholderia multivorans*, contact-dependent growth inhibition, CDI, two-partner secretion systems, interbacterial antagonism, type Vb secretion

## Abstract

Belonging to the two-partner secretion family of proteins, contact-dependent growth inhibition (CDI) systems mediate interbacterial antagonism among closely related Gram-negative bacteria. The toxic portion of a large surface protein, BcpA/CdiA, is delivered to the cytoplasm of neighboring cells where it inhibits growth. Translocation of the antibacterial polypeptide out of the producing cell requires an associated outer membrane transporter, BcpB/CdiB. Some bacteria, including many *Burkholderia* species, encode multiple distinct CDI systems, but whether there is interaction between these systems is largely unknown. Using *Burkholderia cepacia* complex species as a model, here we show that related BcpB transporters exhibit considerable secretion flexibility and can secrete both cognate and non-cognate BcpA substrates. We also identified an additional unique *Burkholderia dolosa* CDI system capable of mediating interbacterial competition and demonstrated that its BcpB transporter has similar relaxed substrate specificity. Our results showed that two BcpB transporters (BcpB-2 and BcpB-3) were able to secrete all four of the *B. dolosa* BcpA toxins, while one transporter (BcpB-1) appeared unable to secrete even its cognate BcpA substrate under the tested conditions. This flexibility provided a competitive advantage, as strains lacking the full repertoire of BcpB proteins had decreased CDI activity. Similar results were obtained in *Burkholderia multivorans*, suggesting that secretion flexibility may be a conserved feature of *Burkholderia* CDI systems. Together these findings suggest that the interaction between distinct CDI systems enhances the efficiency of bacterial antagonism.

**IMPORTANCE:**

The *Burkholderia cepacia* complex (Bcc) is a group of related opportunistic bacterial pathogens that occupy a diverse range of ecological niches and exacerbate disease in patients with underlying conditions. Contact-dependent growth inhibition (CDI) system proteins, produced by Gram-negative bacteria, contain antagonistic properties that allow for intoxication of closely related neighboring bacteria via a secreted protein, BcpA. Multiple unique CDI systems can be found in the same bacterial strain, and here we show that these distinct systems interact in several Bcc species. Our findings suggest that the interaction between CDI system proteins is important for interbacterial toxicity. Understanding the mechanism of interplay between CDI systems provides further insight into the complexity of bacterial antagonism. Moreover, since many bacterial species are predicted to encode multiple CDI systems, this study suggests that interactions between these distinct systems likely contribute to the overall competitive fitness of these species.

## INTRODUCTION

In both environmental niches and infection sites, bacteria often reside in diverse polymicrobial communities, where they engage in competitive and cooperative interactions with other microorganisms. Contact-dependent growth inhibition (CDI) system proteins mediate competition among closely related Gram-negative proteobacteria. In CDI systems, the toxic C-terminus of a large surface-localized exoprotein is delivered to the cytoplasm of a neighboring bacterium upon direct cell-to-cell contact (1). Delivery of the toxic domain, which frequently has nuclease activity, results in cell death or growth arrest of the recipient cells. Auto-inhibition is prevented by the production of a cognate immunity protein that binds to the toxin and blocks its activity. Because protection by immunity proteins is allele specific, exchange of CDI system toxins allows for self vs non-self discrimination (1, 2).

Some organisms encode multiple CDI systems that each containa distinct toxin-immunity pair. In *Escherichia coli*, *Acinetobacter baumannii*, *Pseudomonas aeruginosa,* and several *Burkholderia cepacia* complex (Bcc) species, these systems have been shown to independently mediate interbacterial competition, often displaying differences in gene expression or toxin potency (3 - 7). Whether cross talk may occur among CDI systems produced by the same strain has not been examined.

CDI systems are a subset of two-partner secretion (TPS) pathway (type Vb secretion) proteins, consisting of an outer membrane transporter (the “TpsB” partner) that facilitates the secretion of its cognate exoprotein (“TpsA”) onto the cell surface (8). In CDI systems in *Burkholderia* and related genera, the *bcpA* and *bcpB* genes (termed *cdiA* and *cdiB* in other species) encode the exoprotein and outer membrane transporter, respectively (9, 10). Additional components of *Burkholderia* CDI systems are the immunity protein BcpI and, sometimes, a predicted lipoprotein of unknown function, BcpO.

Research on representative TPS systems has defined a model for the secretion pathway of these proteins. After transport into the periplasm via the Sec machinery, TpsA remains in an unfolded state until the TpsB transporter incorporates TpsA into the outer membrane, and it is progressively folded at the cell surface (11 - 13). The TpsA proteins are large filamentous exoproteins with typical hemagglutinin repeats and a conserved N-terminal TPS domain required for recognition by a TpsB transporter (11, 14). The TpsB transporters are Omp85 superfamily members that consist of an outer membrane-embedded β-barrel channel, an N-terminal α-helix (H1) plug that inserts into the barrel pore, a short periplasmic polypeptide linker, and two periplasmic polypeptide transport-associated (POTRA) domains (15 - 17). The POTRA domains interact with the TPS domain on the TpsA protein and are necessary for substrate recognition and secretion (18, 19).

The specificity of a TpsB transporter for its cognate TpsA partner varies between systems. Many TpsB transporters can secrete only their cognate partner, while other transporters can secrete more than one TpsA effector (20). The CdiB transporters from *A. baumannii* ACICU and *E. coli* EC93, which share ~23% amino acid sequence identity, are not interchangeable and specifically secrete their cognate CdiA proteins (21). However, little is known about the specificity of more closely related BcpB or CdiB transporters, such as those that would be produced by an organism with multiple CDI systems.

Here, we use *B. cepacia* complex species that each produced multiple distinct CDI systems to examine the specificity of BcpB transporters for BcpA toxin secretion. The results show that even though each complete CDI system includes an associated BcpB, the transporters display a high degree of promiscuity and generally secrete both cognate and non-cognate BcpA proteins efficiently. While three BcpB proteins in *Burkholderia dolosa* each secreted multiple BcpA toxins, differences in gene expression appeared to limit which transporters were available. We also report that the relaxed specificity of BcpB proteins extends to *Burkholderia multivorans,* suggesting that interaction of non-cognate BcpB-BcpA pairs may be a common characteristic of bacterial species that produce multiple CDI systems.

## RESULTS

### *B. dolosa* AU0158 contains an additional putative CDI system

**Fig 1:**
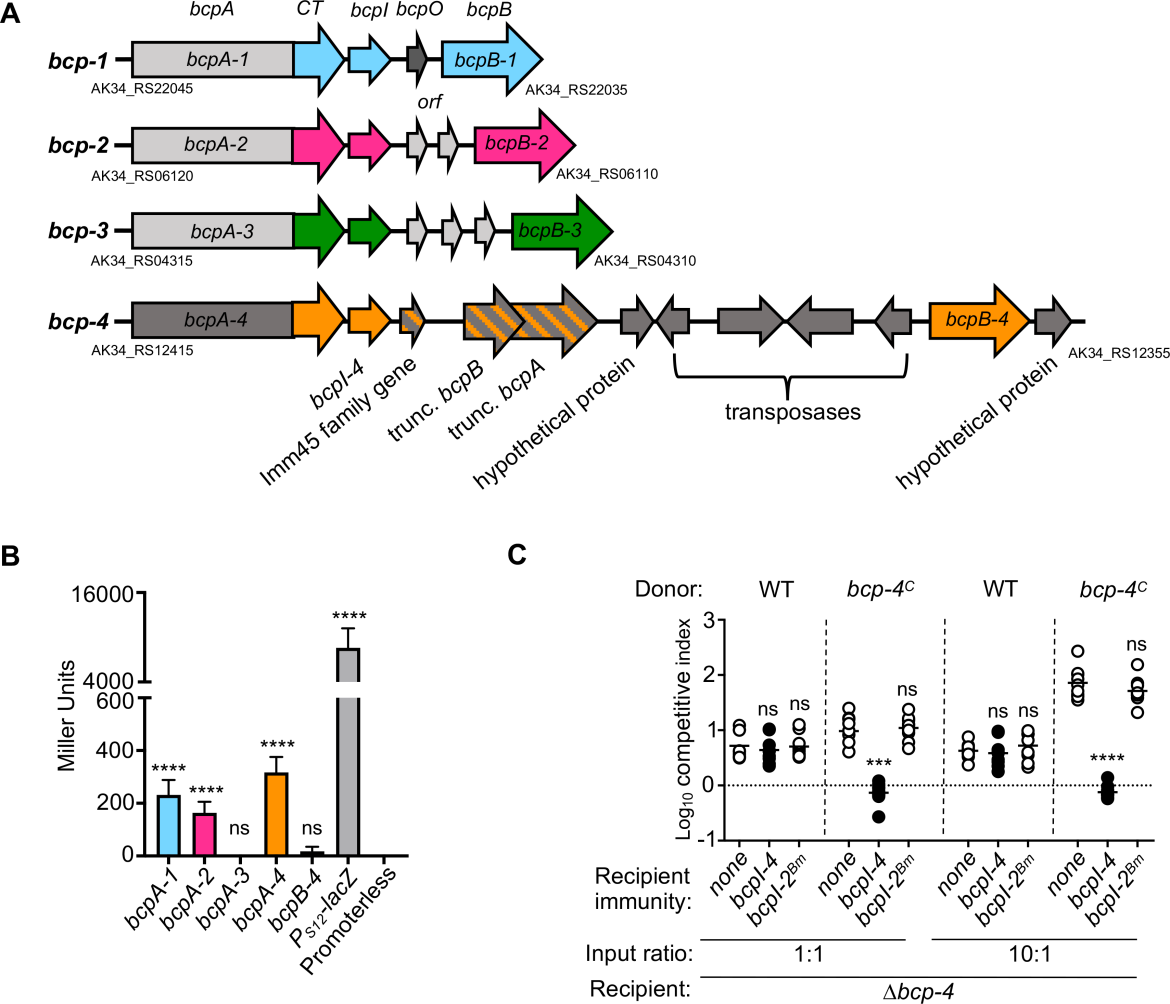
Activity of putative *B. dolosa* CDI system-4. (**A**) Diagram of the *Bd*AU0158 *bcp-1*, *bcp-2*, *bcp-3,* and *bcp-4* loci. Truncated *bcp* genes are located 3′ to *bcpI-4* and are indicated by slashes. Non-*bcp* genes associated with *bcp-4* (dark gray) encode hypothetical proteins and putative transposases. (**B**) Beta-galactosidase assay of *lacZ* reporters for putative promoters of the *Bd*AU0158 *bcpA-1*, *bcpA-2*, *bcpA-3, bcpA-4*, or *bcpB-4* genes and control reporters P_S12_-*lacZ* (constitutive) and promoterless *lacZ*. Bars show the mean of miller units from three independent experiments, each with three replicates. (**C**) Interbacterial competition assays between *Bd*AU0158 wild type (WT) or constitutively expressed *bcpA-4* (*bcp-4^C^*) donor bacteria and *∆bcp-4* recipient bacteria that were complemented with the cognate *bcpI-4, bcpI-2* from *B. multivorans* CGD2M (*bcpI-2^Bm^),* or no *bcpI* (none). Symbols represent log_10_ competitive index values (ratio of donor to recipient) from three independent experiments, and bars show the means (*n* = 9). Competition assays were performed at a 1:1 or 10:1 (donor to recipient) ratio as indicated. Dashed line shows log_10_ competitive index = 0 (no competition). ns, not significant; ***P < 0.001; and ****P < 0.0001; compared to WT donor cells competed against no immunity (none) recipient cells in each panel for competition assays or promoterless reporter for the beta-galactosidase assay.

*B. dolosa* strain AU0158 (*Bd*AU0158) was shown to produce three unique CDI systems capable of mediating interbacterial competition, but only system-1 and system-2 were expressed in laboratory conditions (6). Each of the three CDI systems encodes a distinct BcpB transporter, sharing ~80% amino acid identity. Additionally, we identified a fourth *bcpB* gene downstream of a cryptic *bcp* locus (referred to as *bcp-4*) located on *Bd*AU0158 chromosome 3 (Fig. 1A). The *bcp-4* region resembles other loci that encode *Burkholderia*-type CDI systems, with the gene order *bcpAI(O)B*. However, the distance between the *bcpI* and *bcpB* genes is ~8,000 bp, a gap larger than what is typically found for *Burkholderia*-type CDI loci. The *bcp-4* locus has multiple open reading frames (ORFs) between the *bcpI* and *bcpB genes,* although none of these ORFs are predicted to encode a BcpO lipoprotein. Instead, many of the ORFs are predicted to encode transposases, integrases, or genes that produce uncharacterized hypothetical proteins. Interestingly, immediately downstream of *bcpAI-4* are additional *bcp-*like genes: an ORF annotated to encode an immunity 45 family protein and truncated *bcpB* and *bcpA* genes. Despite the chromosomal distance between the *bcpAI-4* and *bcpB-4* genes, the *bcp-4* region contains the genetic components necessary to produce a CDI system.

### Putative *Bd*AU0158 *bcpA-4* promoter is active under *in vitro* competition conditions

To examine the expression of the *bcp-4* genes, ~500 bp 5′ to the *bcpA-4* and *bcpB-4* translational start sites were fused to promoterless *lacZ* genes and delivered to *att*Tn7 sites of *Bd*AU0158. The resulting reporter strains were compared to similar reporter strains generated for the other three *Bd*AU0158 *bcpA* genes (6). When grown in monoculture under the same conditions as those used for competition experiments, the *bcpA-1* and *bcpA-2* reporters showed low levels of β-galactosidase activity (Fig. 1B). By contrast, P*_bcpA-3_-lacZ* showed no detectable activity (Fig. 1B), as previously demonstrated (6). The P*_bcpA-4_-lacZ* reporter also showed low levels of β-galactosidase activity, while the P*_bcpB-4_-lacZ* activity levels did not significantly differ from the promoterless control. These data suggest that *bcpA-4* is expressed and, therefore, may produce a functional CDI system protein, while *bcpB-4* is likely not expressed under the conditions tested.

### *Bd*AU0158 *bcp-4* encodes a functional CDI system

To determine the functionality of the *Bd*AU0158 *bcp-4* CDI system, a mutant strain containing an unmarked, in-frame deletion of *bcpA-4* through *bcpB-4* was generated (*∆bcp-4*). When wild-type donor cells were competed against the *∆bcp-4* recipient cells at a 1:1 or 10:1 ratio, there was no difference in the competitive index (CI) between non-immune recipient cells or those producing cognate immunity protein BcpI-4 (Fig. 1C). These data suggest that the *Bd*AU0158 BcpAIB-4 CDI system does not mediate interbacterial competition under conditions of native expression *in vitro*.

We hypothesized that native expression of *bcpA-4* is not sufficient for CDI-mediated competition and constructed a strain in which the putative *bcpA-4* promoter was replaced with the strong, constitutively active *Burkholderia thailandensis rpsL* (ribosomal S12 subunit) promoter, resulting in strain *bcp-4^C^*. Following co-culture, the *Bd*AU0158 *bcp-4^C^* donor bacteria outcompeted the *∆bcp-4* mutant by ~10-fold when inoculated at a 1:1 ratio and by ~100-fold when inoculated at a 10:1 ratio (Fig. 1C). Introduction of the cognate *bcpI-4* immunity gene protected recipient cells from *bcpA-4*-mediated killing. As expected, complementation with a gene encoding a heterologous immunity protein from *B. multivorans* (*Bm*CGD2M *bcpI-2*) did not provide protection against CDI, indicating that *bcpI-4* protection was allele specific. These results indicate that the *bcp-4* locus encodes a functional CDI system that can mediate interbacterial competition when expressed at a high level.

### Cognate BcpB transporters are not required for *B. dolosa* CDI-mediated competition

**Fig 2:**
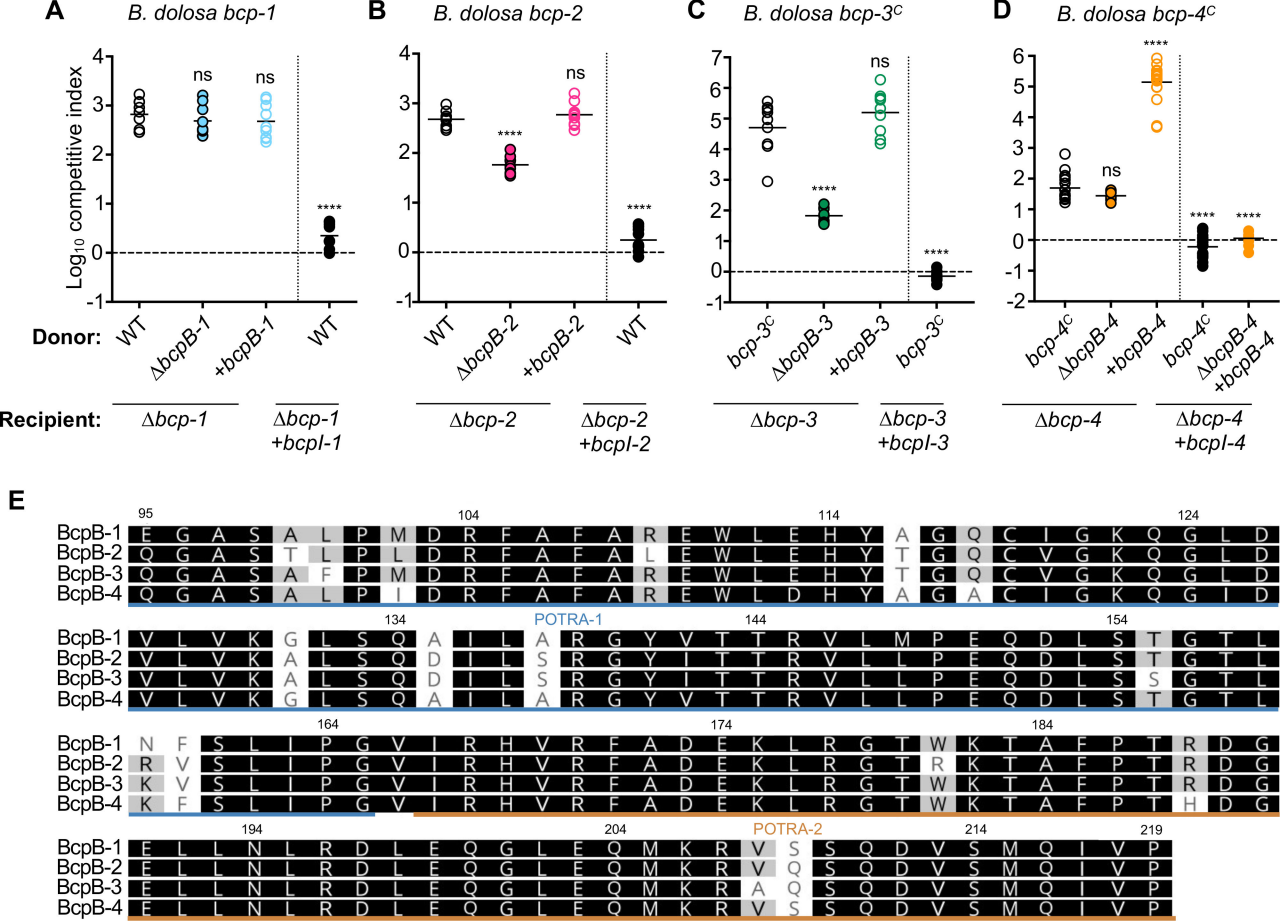
Role of cognate BcpB transporters in BcpA-mediated interbacterial antagonism. (**A**) Interbacterial competition assays between the indicated donor cells: *Bd*AU0158 wild type (WT; open circles), *∆bcpB-1* (closed blue circles), or *∆bcpB-1* complemented with P_S12_-*bcpB-1* at an *att*Tn7 site (+*bcpB-1*; open blue circles) and recipient cells: *∆bcp-1* or *∆bcp-1* complemented with cognate *bcpI-1*. (**B**) Interbacterial competition assays between the indicated donor cells: *Bd*AU0158 wild type (open circles), *∆bcpB-2* (closed pink circles), or *∆bcpB-2* complemented with P_S12_-*bcpB-2* at an *att*Tn7 site (+*bcpB-2*; open pink circles) and recipient cells: *∆bcp-2* or *∆bcp-2* complemented with cognate *bcpI-2*. (**C**) Interbacterial competition assays between donor bacteria constitutively expressing: *Bd*AU0158 *bcpAIOB-3* (*bcpA-3^C^)* (parent; open circles), *bcpA-3^C^ ∆bcpB-3* (closed green circles), or *bcpA-3^C^ ∆bcpB-3* complemented with P_S12_-*bcpB-3* at an *att*Tn7 site (+*bcpB-3*; open green circles) and recipient cells: *∆bcp-3* or *∆bcp-3* complemented with cognate *bcpI-3*. (**D**) Interbacterial competition assays between the donor cells that constitutively express: *Bd*AU0158 *bcpA-4* (*bcpA-4^C^*) (parent; open circles), *bcpA-4^C^ ∆bcpB-4* (closed orange circles), or *bcpA-4^C^ ∆bcpB-4* complemented with P_S12_-*bcpB-4* at an *att*Tn7 site (+*bcpB-4*; open orange circles) and the indicated recipient cells: *∆bcp-4* or *∆bcp-4* complemented with cognate *bcpI-4* immunity. Competitive Index (CI) for competition assays were calculated as (output donor CFU/recipient CFU)/(input donor CFU/recipient CFU). Symbols represent log_10_ CI values from three independent experiments, and horizontal bar shows means (*n* = 9–18). Competition assays in panel D were performed at a 10:1 ratio. Dashed line shows log_10_ competitive index = 0 (no competition). ns, not significant and ****P < 0.0001; compared to WT donor cells competed against no immunity recipient cells. (**E**) Amino acid alignments of *Bd*AU0158 BcpB-1, BcpB-2, BcpB-3, and BcpB-4 polypeptide transport-associated domains. Similarity is denoted by grayscale; residues similar in all sequences are highlighted in black, and residues similar in 50% of sequences are highlighted in gray. Regions underlined in blue or orange represent POTRA-1 or POTRA-2 domains, respectively.

Many bacterial species encode multiple CDI systems within the same strain. Since *B. dolosa* AU0158 contains four unique CDI systems, this strain provides a useful model for investigating potential interplay between distinct CDI systems. To examine the specificity of *Bd*AU0158 BcpB transporters, strains containing unmarked, in-frame deletion mutations of each of the *bcpB* genes were generated, resulting in *∆bcpB-1*, *∆bcpB-2, ∆bcpB-3,* and *∆bcpB-4* mutants. Interbacterial competition assays between these *bcpB* mutants and the corresponding immunity-deficient (*∆bcpAIOB*) recipient cells were used to determine if each BcpB protein is required for the secretion of its cognate BcpA toxin. Surprisingly, the *∆bcpB-1* mutant inhibited the *∆bcp-1* recipient strain similarly to wild-type donors, implying that wild-type levels of BcpA-1 were still secreted and delivered to recipient bacteria in the absence of BcpB-1 (Fig. 2A). The *∆bcpB-2* mutant was also able to outcompete susceptible recipient cells but showed a ~10-fold defect in competitive index as compared to wild-type donor bacteria (Fig. 2B). Interbacterial killing was restored to wild-type levels when donor cells were complemented with *bcpB-2* at a neutral chromosomal site. Thus, BcpB-2 is also unnecessary for secretion of its cognate BcpA protein but does appear to participate in BcpA-2 secretion.

Because *bcpA-3* is not expressed under laboratory conditions, competitions investigating this toxin were conducted in strains that constitutively expressed *bcpA-3* due to replacement of the native *bcpA-3* promoter with P_S12_(*bcp-3^C^*), as previously described (6). The *bcp-3^C^ ∆bcpB-3* mutant outcompeted *∆bcp-3* recipient cells, although the CDI activity was ~100-fold less than for the *bcp-3^C^* parent strain (Fig. 2C). These results show that BcpB-3 contributes to but is not required for cognate BcpA-3 secretion.

The *bcp-4* overexpression strain (*bcp-4^C^*, Fig. 1C) was also used here to test the contribution of *bcpB-4*. Similar to *bcpB-1*, there was no defect in growth inhibition of *∆bcp-4* recipient bacteria by donor cells lacking *bcpB-4* (Fig. 2D), indicating that BcpB-4 is not required for BcpA-4 secretion. However, complementation of the *∆bcpB-4* mutant with overexpressed *bcpB-4* resulted in a high level of BcpA-4-mediated CDI, representing a ~1,000-fold increase in competitive index as compared to the *bcp-4^C^* parent or *∆bcpB-4* mutant strains. This high level of CDI activity was eliminated when the complemented donor strain (*bcp-4^C^ ∆bcpB-4+bcpB-4*) was competed against recipient cells supplemented with cognate *bcpI-4* immunity. These data show that BcpB-4 is not necessary for BcpA-4 secretion but suggest that the transporter can secrete the toxin when it is overproduced.

Altogether, these data indicate that the four BcpA toxins still mediate CDI in the absence of their cognate BcpB transporters. Because the percent identity among the *Bd*AU0158 BcpB polypeptide transport-associated domains, POTRA-1 and POTRA-2 are 74% and 93%, respectively (Fig. 2E; Fig. S1), we hypothesized that BcpA proteins could be secreted by non-cognate BcpB transporters.

### Specificity of BcpB transporters for BcpA TPS domains

**Fig 3:**
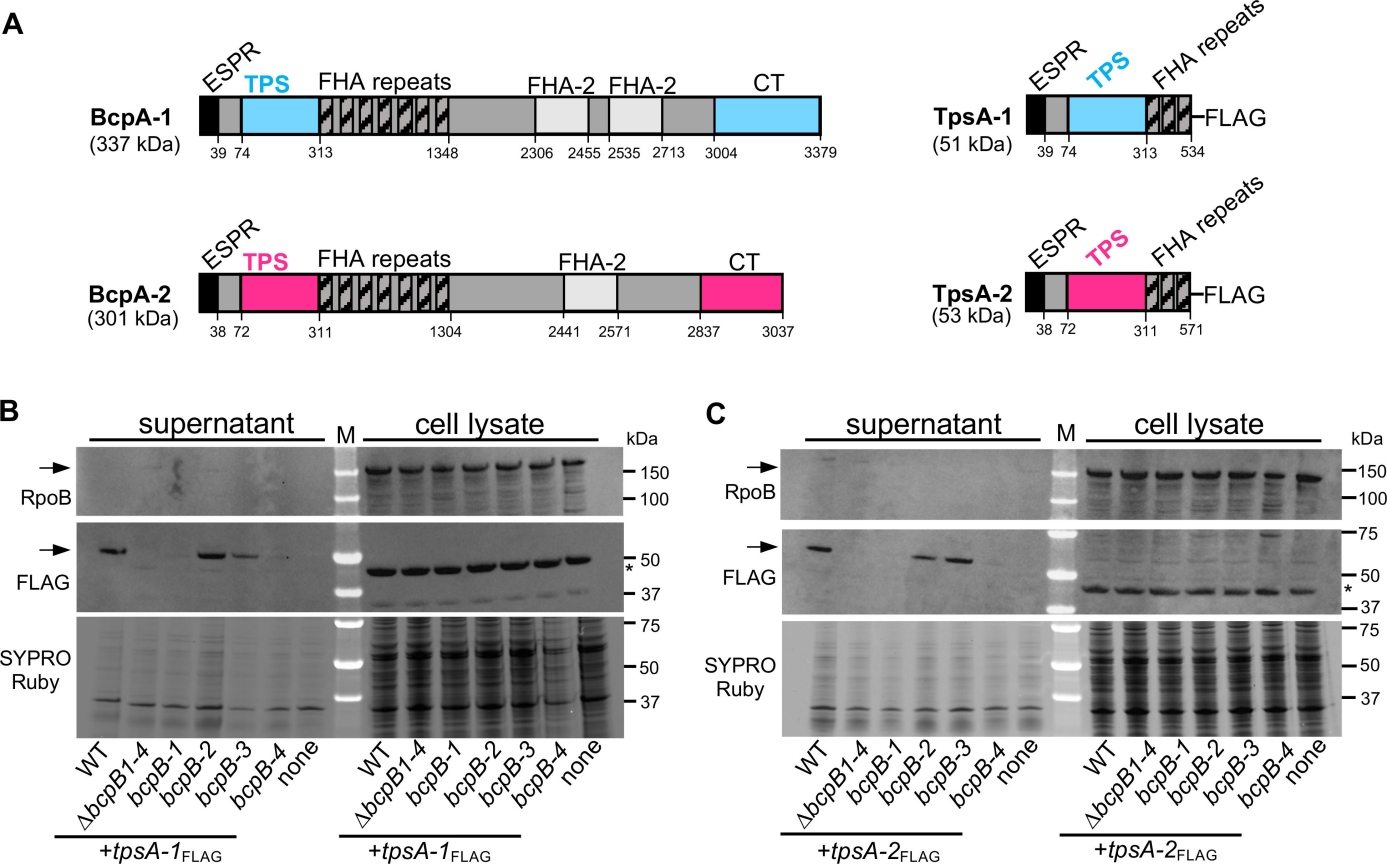
Secretion of truncated BcpA polypeptides by BcpB transporters. (**A**) Graphic representation of the BcpA-1 and BcpA-2 proteins (left) and corresponding TpsA-1 and TpsA-2 polypeptides (right). FHA repeats identified as FHA β helical repeats, ESPR classified as extended signal peptide of Type V secretion systems, and FHA-2 identified as Fil_Haemagg_2 by NCBI Conserved Domain Database. (**B and C**) Western blots of concentrated culture supernatants and whole cell lysate of wild type (WT), quadruple ∆*bcpB-1* ∆*bcpB-2* ∆*bcpB-3* ∆*bcpB-4* mutant (*∆B1-4*), triple mutants containing one natively expressed *bcpB* gene: *∆bcpB-2 ∆bcpB-3 ∆bcpB-4* (*bcpB-1*), *∆bcpB-1 ∆bcpB-3 ∆bcpB-4* (*bcpB-2*), *∆bcpB-1 ∆bcpB-2 ∆bcpB-4* (*bcpB-3*), and *∆bcpB-1 ∆bcpB-2 ∆bcpB-3* (*bcpB-4*) complemented with either (**B**) FLAG-tagged BcpA-1 TPS (*tpsA-1*) or (**C**) FLAG-tagged BcpA-2 TPS (*tpsA-2*). Wild-type bacteria that lack a *tpsA* construct (none) were used as a negative FLAG control. Equal protein amounts for each fraction (supernatant and cell lysate) were resolved on SDS-PAGE gels. Panels have shown blotting with anti-*E*. *coli* RNA polymerase β subunit (RpoB, top) or anti-FLAG peptide (middle) antibodies and total protein visualization by SYPRO Ruby-stained gels (bottom). Expected masses for TpsA-1, TpsA-2, and RpoB are ~53, ~56, and 150 kDa, respectively. Arrows show TpsA-FLAG or RpoB bands, and asterisks indicate non-specific bands.

It has been previously shown for other TPS systems that a truncated TpsA protein consisting of the signal peptide and the TPS domain is efficiently secreted into the culture supernatant in a TpsB-dependent manner (18, 20, 22, 23). To directly examine the secretion of the BcpA proteins, similar TpsA constructs were created for the two proteins that mediate CDI under laboratory conditions, BcpA-1 and BcpA-2. These genetic constructs encoded C-terminally truncated BcpAs encompassing the signal peptide, predicted TPS domain, a portion of the FHA β helical repeat domains, and a FLAG epitope tag (Fig. 3A). To determine the role each BcpB transporter plays in BcpA secretion, a quadruple *∆bcpB* mutant lacking all four transporters (*∆bcpB1-4*) and a series of triple *∆bcpB* deletion mutants that each contained only one natively expressed transporter were constructed. The two constructs encoding truncated BcpA-1 and BcpA-2 proteins, termed *tpsA-1* and *tpsA-2*, were each delivered in single copy to a neutral chromosome site in these mutant strains.

TpsA-FLAG proteins of the expected size (~50 kDa) were only detected in culture supernatants (Fig. 3B and C; Fig. S2). As expected, TpsA-1 and TpsA-2 were not detected in the *∆bcpB1-4* mutant or in the wild-type strain lacking *tpsA* constructs. Both TpsA-1 (Fig. 3B) and TpsA-2(Fig. 3C) were detected in supernatants when they were produced in wild-type bacteria or the triple *bcpB* mutants containing *bcpB-2* or *bcpB-3* alone. Low levels of TpsA-1 were sometimes observed above the limit of detection in the culture supernatant of the strains containing *bcpB-1* or *bcpB-4* (Fig. 3B; Fig. S2D). The non-secreted TpsA-1 and TpsA-2 did not accumulate in the cytoplasm or insoluble (membrane) fractions but appeared to be degraded (Fig. S2A).

Overall, these results indicate that truncated BcpA polypeptides are produced and secreted into the culture medium in a BcpB-dependent manner, primarily by BcpB-2 and BcpB-3. This indicates that the domains contained on these proteins are sufficient for BcpA secretion, which is consistent with observations in other TPS and CDI systems (19 - 21). Furthermore, these results support our previous findings that secretion of BcpA-1 and BcpA-2 is not dependent upon the cognate BcpB transporter.

### BcpB-2 and BcpB-3 transporters can secrete all four BcpA toxins

**Fig 4:**
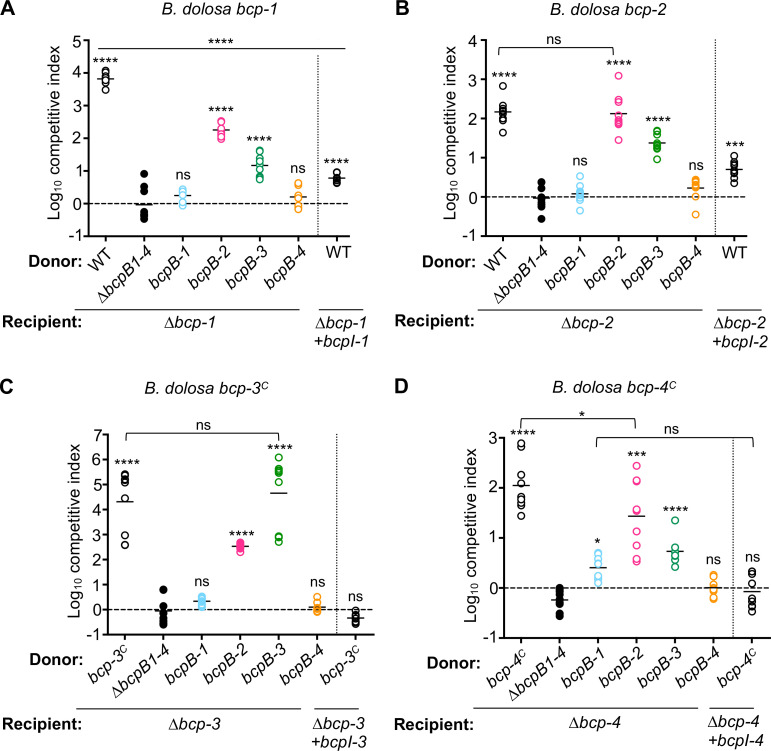
Contribution of natively expressed BcpB transporters during *Bd*AU0158 CDI-mediated competition. Interbacterial competition assays between *Bd*AU0158 wild type (WT; open circles) or donor cells that each contain a single chromosomal copy of the natively expressed *bcpB* indicated: ∆*bcpB-1* ∆*bcpB-2* ∆*bcpB-3* ∆*bcpB-4 (∆bcpB1-4;* closed circles), *∆bcpB-2 ∆bcpB-3 ∆bcpB-4* (*bcpB-1*; blue circles), *∆bcpB-1 ∆bcpB-3 ∆bcpB-4* (*bcpB-2*; pink circles), *∆bcpB-1 ∆bcpB-2 ∆bcpB-4* (*bcpB-3*; green circles), *∆bcpB-1 ∆bcpB-2 ∆bcpB-3* (*bcpB-4*; orange circles), and the indicated recipient cells: (**A**) *∆bcp-1* or *∆bcp-1* complemented with cognate *bcpI-1,* (**B**) *∆bcp-2* or *∆bcp-2* complemented with cognate *bcpI-2,* (**C**) *∆bcp-3* or *∆bcp-3* complemented with cognate *bcpI-3,* and (D) *∆bcp-4* or *∆bcp-4* complemented with cognate *bcpI-4*. For the donor cells in panels (**C**) and (**D**), the *bcpA-3* and *bcpA-4* promoters were replaced with the P_S12_ constitutive promoter to generate *bcp-3^C^* and *bcp-4^C^* parent strains, respectively. Symbols represent log_10_ competitive index values (ratio of donor to recipient) from three independent experiments, and bars show the mean (*n* = 9). Experiments in (**D**) were performed at a 10:1 (donor to recipient) ratio. Dashed line shows log_10_ competitive index = 0 (no competition). ns, not significant; *P < 0.05; ***P < 0.001; and ****P < 0.0001 compared to *∆bcpB1-4* donor cells, unless indicated by line or brackets.

Our findings suggest that both cognate and non-cognate BcpB transporters participate in BcpA secretion in *B. dolosa*. To determine the role each BcpB transporter plays in BcpA toxin secretion and delivery, we used the triple *∆bcpB* mutants to individually examine the activity of one natively expressed transporter at a time. These *bcpB* deletion mutants were competed against a series of recipient cells that each lacked one CDI system (thus lacking immunity to only one BcpA protein). As expected, the quadruple *∆bcpB1-4* mutant did not outcompete any recipient strain as it lacks all BcpB transporters (Fig. 4).

Only the donor strains containing *bcpB-2* or *bcpB-3* outcompeted a *∆bcp-1* recipient, indicating that BcpB-2 and BcpB-3 each secreted BcpA-1 toxin that was capable of mediating CDI (Fig. 4A). However, these competitive indices were significantly less than those of wild-type donors, suggesting that production of only one BcpB transporter is not sufficient for maximum BcpA-1-mediated killing. The donor strains containing only *bcpB-1* or *bcpB-4* did not outcompete *∆bcp-1* recipient cells, suggesting that BcpA-1 was not secreted by natively produced BcpB-1 or BcpB-4 or that BcpA-1 secreted by these transporters was unable to mediate CDI. While Fig. 1A indicated that BcpB-1 is not required for BcpA-1 secretion, these results further suggest that BcpB-1 does not participate in the secretion of its cognate BcpA protein under these conditions.

Similar results supporting the importance of BcpB-2 and BcpB-3 were also found for the remaining BcpA proteins. CDI activity against *∆bcp-2* or *∆bcp-3* mutant recipients was only observed for donor strains producing BcpB-2 or BcpB-3 (Fig. 4B and C). In each case, donor strains producing the cognate BcpB transporter outcompeted recipient bacteria at levels similar to wild-type donors. Donor bacteria producing the non-cognate transporter (either BcpB-2 or BcpB-3) also outcompeted recipient cells but at levels less than wild type. These data suggest that BcpA-2 and BcpA-3 can be secreted by multiple BcpB transporters but may prefer their cognate transporters.

BcpA-4 also appeared to utilize BcpB-2 and BcpB-3, but the competitive indices for these mutant co-cultures were significantly less than for the parent strain (Fig. 4D). While not definitive, this result suggests that production of either BcpB-2 or BcpB-3 may not be sufficient for maximum BcpA-4-mediated killing. BcpA-4 activity was not observed when secretion depended on BcpB-4, likely due to poor *bcpB-4* expression under these conditions (Fig. 1B). Moreover, previous data showed that *bcpB-4* overexpression led to increased CDI by BcpA-4 (Fig. 2D), implying that BcpA-4 can be efficiently secreted by its cognate transporter. By contrast, overexpression of *bcpB-1* did not affect BcpA-1-mediated CDI (Fig. 2A), suggesting that low *bcpB-1* expression may not explain the lack of CDI activity by donor bacteria that only contain *bcpB-1*.

Together these findings indicate that *B. dolosa* BcpA toxins mediate CDI activity when secreted from both cognate and non-cognate BcpB, but the toxins vary in their specificity for the transporters. All four BcpA proteins were secreted from strains containing either *bcpB-2* or *bcpB-3*, but none of the toxins mediated CDI when only *bcpB-1* or *bcpB-4* was present. An implication of this result is that BcpB-3 must be produced and active, even though the *bcpA-3* promoter is inactive under these conditions (6). Thus, the activities of this cognate BcpA/BcpB pair are uncoupled in *B. dolosa*.

These results are also generally consistent with the TpsA-1 and TpsA-2 secretion assays, which showed secretion primarily by BcpB-2 and BcpB-3 (Fig. 3). While we cannot rule out differences in the secretion of truncated BcpA polypeptides (“TpsA-1” and “TpsA-2”) as compared to full-length BcpAs, it is likely that the occasional low level of TpsA secretion detected for BcpB-1 and/or BcpB-4 (Fig. 3; Fig. S2) was insufficient to cause measurable CDI.

### Competition between BcpA-1 and BcpA-2 for secretion by BcpB-3

Since our data indicate that multiple BcpA toxins are secreted by BcpB-2 and BcpB-3, we next sought to determine whether competition occurs for secretion by the available BcpB transporters. To do this, we compared the CDI activities of donor strains that utilized only BcpB-3 but had varying levels of potentially competing BcpA-1 and BcpA-2 proteins. This allowed us to ask whether secretion through a single transporter, BcpB-3, was impacted by levels of substate BcpA proteins. When examining BcpA-1 activity, we asked whether interbacterial killing was impacted by BcpA-2 levels, and vice versa.

**Fig 5:**
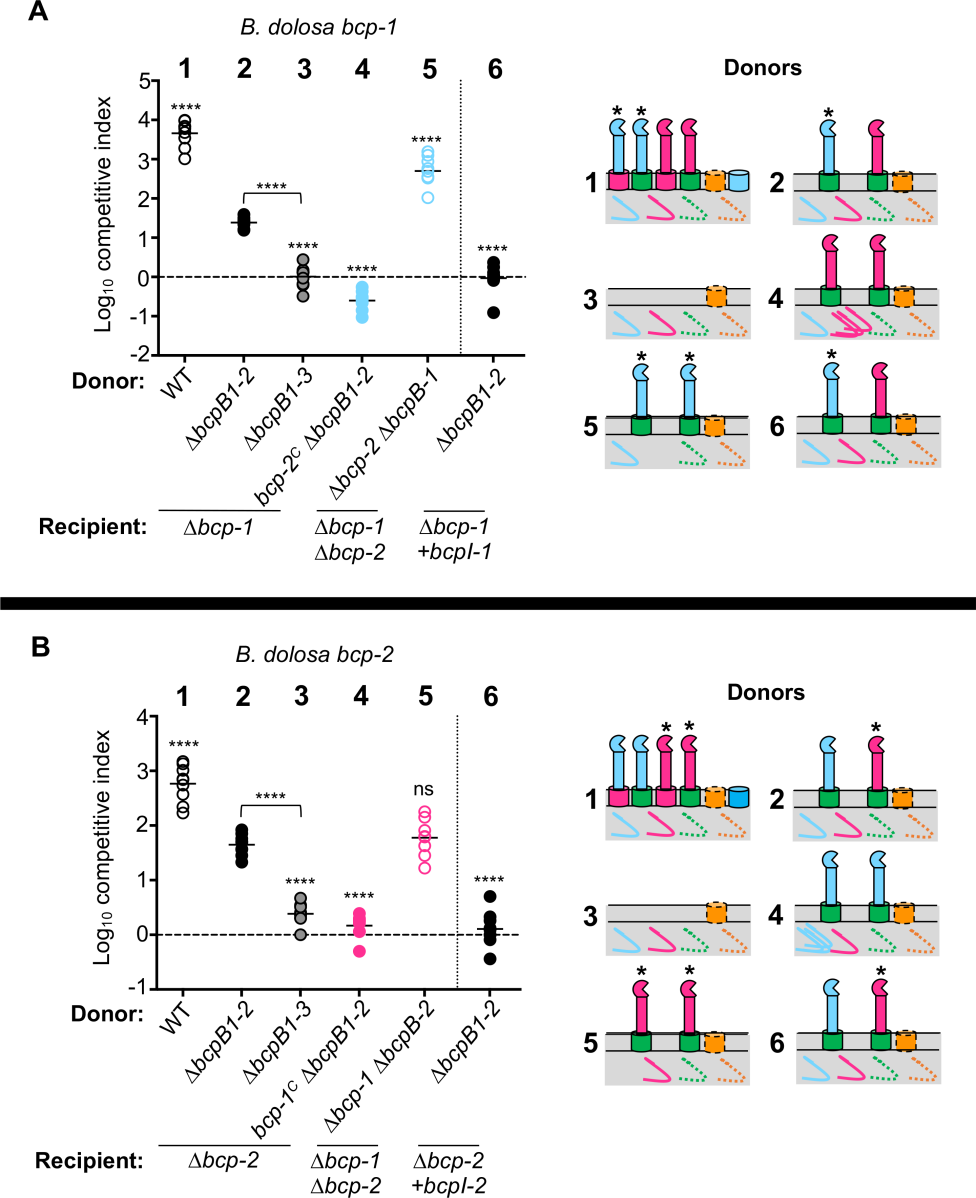
Competition among BcpA toxins for secretion by the BcpB-3 transporter. (**A**) Interbacterial competition assays (left) between the indicated donor cells: *Bd*AU0158 wild type (WT; closed black circles), *∆bcpB-1 ∆bcpB-2* (*∆bcpB1-2*; closed black circles), *∆bcpB-1 ∆bcpB-2 ∆bcpB-3 (∆bcpB1-3;* closed gray circles), *∆bcpB1-2* mutant that overexpresses *bcpAIOB-2* (*bcp-2^C^ ∆bcpB-1–2*; closed blue circles), and *∆bcp-2 ∆bcpB-1* (open blue circles) and the indicated recipient cells: *∆bcp-1, ∆bcp-1 ∆bcp-2,* or *∆bcp1* complemented with cognate *bcpI-1*. (Right) Simplified cartoon model to illustrate donor cells used in the assay (numbers correspond to co-cultures numbered above graph). Barrels represent OM BcpB proteins. Curved lines represent periplasmic BcpA that is secreted by the indicated BcpB proteins (“stick-pacman” shapes are secreted BcpA). Blue, BcpAB-1; pink, BcpAB-2; green, BcpAB-3; orange, BcpAB-4. Asterisks highlight that BcpA-1 activity is measured in this assay. (**B**) Interbacterial competition assays (left) between the indicated donor cells *Bd*AU0158 wild type (open black circles), *∆bcpB-1 ∆bcpB-2* (*∆bcpB1-2*; closed black circles), *∆bcpB-1 ∆bcpB-2 ∆bcpB-3 (∆bcpB1-3;* closed gray circles), *∆bcpB1-2* mutant that overexpresses *bcpAIOB-1* (*bcp-1^C^ ∆bcpB-2*; closed pink circles), and *∆bcp-1 ∆bcpB-2* (open pink circles) and the indicated recipient cells: *∆bcp-2, ∆bcp-1 ∆bcp-2,* or *∆bcp2* complemented with cognate *bcpI-2*. Symbols represent log_10_ competitive index values (ratio of donor to recipient) from three independent experiments, and bars show the mean (*n* = 9). Dashed line shows log_10_ competitive index = 0 (no competition). ns, not significant; and ****P < 0.0001 compared *∆bcpB1-2* donor cells vs *∆bcp-1* or *∆bcp-2* recipient cells, unless indicated by brackets. (Right) Simplified cartoon model to illustrate donor cells used in the assay, as in (**A**).

These experiments utilized a *∆bcpB-1 ∆bcpB-2* double mutant that depends on BcpB-3 for secretion of BcpA-1 and BcpA-2. Since this double mutant lacks BcpB-2, it outcompeted both *∆bcp-1* (Fig. 5A) and *∆bcp-2* (Fig. 5B) recipient cells to a lesser extent than wild-type donors (Fig. 5A and B, panels 1 and 2). Elimination of BcpB-3 from this mutant (by testing a triple *∆bcpB-1 ∆bcpB-2 ∆bcpB-3* mutant) abolished interbacterial killing of both recipient strains, indicating that the double mutant indeed depended on BcpB-3 for secretion of BcpA-1 and BcpA-2 (Fig. 5A and B, panel 3). Interestingly, when *bcpA-2* was overexpressed, the BcpA-1-mediated CDI activity of the donor cells was eliminated completely (Fig. 5A, panel 4). Similarly, overexpression of *bcpA-1* prevented BcpA-2-mediated CDI activity (Fig. 5B, panel 4). These results indicate that high levels of one BcpA protein can negatively impact the activity of other BcpA proteins when BcpB transporters are limited, likely by competing for secretion by BcpB.

Consistent with this hypothesis, a donor strain lacking *bcpA-2* (*∆bcp-2 ∆bcpB-1*) showed significantly higher levels of BcpA-1-mediated CDI (Fig. 5A, panel 5). However, the reciprocal was not true. Loss of *bcpA-1* did not alter interbacterial killing by BcpA-2 (Fig. 5B, panel 5). These data suggest that BcpA-2 secretion is less sensitive to the presence of BcpA-1 when both BcpA proteins are utilizing a single BcpB transporter, implying that the BcpB-3 transporter may have a higher affinity for BcpA-2 toxin. Altogether, these data indicate that there can be a competition among BcpA proteins for secretion by limited BcpB transporters, and the transporters likely have a higher affinity for specific BcpA toxins.

### Replacing the BcpB-1 POTRA domains changes the functionality of BcpB-1

**Fig 6:**
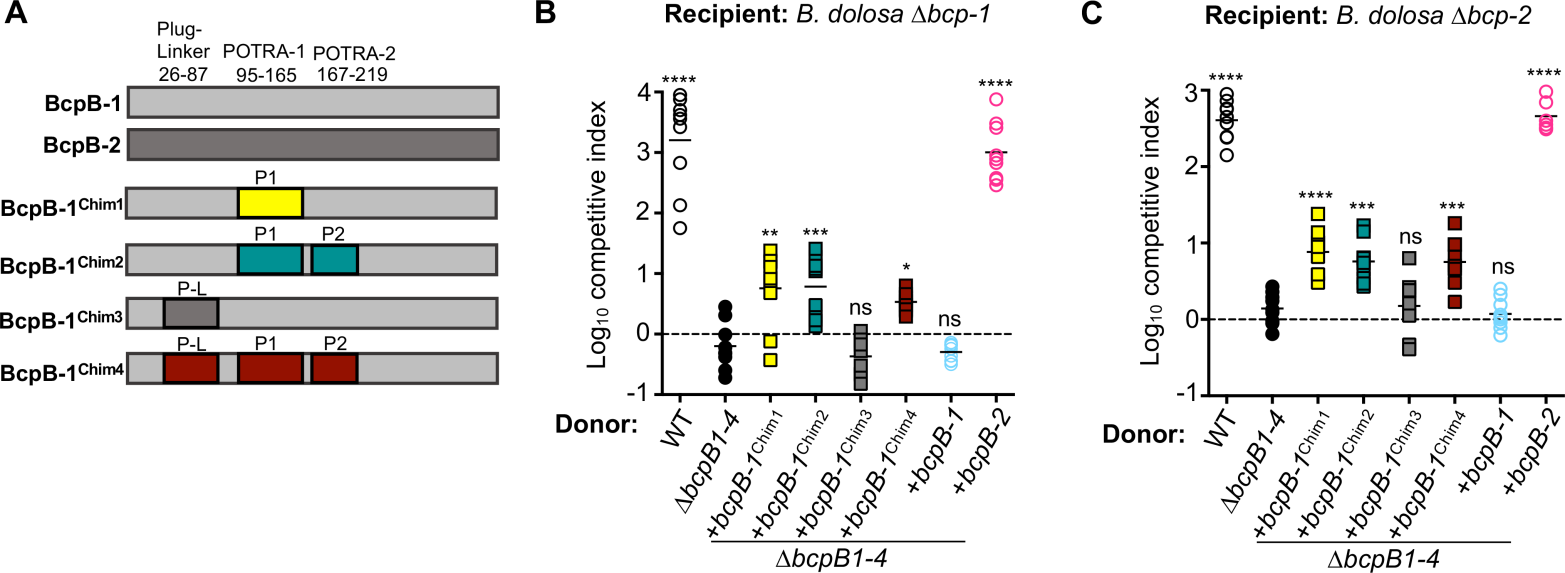
Contribution of BcpB-1 POTRA domains to the secretion of BcpA toxins. (**A**) Graphical representation of the BcpB-1 and BcpB-2 proteins from *B. dolosa* and the four BcpB-1 chimeras. Residue numbers refer to Plug-linker, POTRA-1, or POTRA-2 domains used to construct the chimeras. (**B and C**) Interbacterial competition assays between the indicated donor cells: *Bd*AU0158 wild type (WT; open black circles), ∆*bcpB-1* ∆*bcpB-2* ∆*bcpB-3* ∆*bcpB-4* (*∆bcpB1-4*; closed black circles), and ∆*bcpB1-4* complemented with overexpressed *bcpB-1* (+*bcpB-1^Bd^*; open blue circles), *bcpB-2* (+*bcpB-2^Bd^*; open pink circles), or genes to produce BcpB-1 chimeras Chim1 (+*bcpB-1*^Chim1^; closed yellow squares), Chim2 (+*bcpB-1*^Chim2^; closed teal squares), Chim3 (+*bcpB-1*^Chim3^; closed gray squares), or Chim4 (+*bcpB-1*^Chim4^; closed red squares), and *Bd*AU0158 (**B**) ∆*bcp-1* recipient cells or (**C**) ∆*bcp-2* recipient cells. Symbols represent log_10_ CI values from three independent experiments, and horizontal bars show the mean (*n* = 6 or 9). Dashed line shows log_10_ competitive index = 0 (no competition). ns, not significant; *P < 0.05; **P < 0.005; ***P < 0.001; and ****P < 0.0001; compared to *∆bcpB1-4* mutant donor cells.

Previous data showed that natively expressed *bcpB-1* does not allow donor cells to inhibit recipient cell growth even with cognate BcpA-1. To determine whether insufficient *bcpB-1* gene expression contributes to this defect, we overexpressed *bcpB-1* in the quadruple *∆bcpB1-4* mutant that does not contain any native *bcpB* genes. While bacteria that overproduced BcpB-2 secreted BcpA-1 (Fig. 6B) and BcpA-2 (Fig. 6C), donor bacteria overexpressing wild-type *bcpB-1* did not show any interbacterial toxicity. Thus, even overproduced BcpB-1 is defective for some step of BcpA secretion or delivery to recipient cells.

To further investigate the apparent lack of BcpB-1 functionality, we utilized chimeric BcpB proteins. For TPS systems in some *Neisseria* species, swapping the POTRA domains can change TpsB specificity (19). To test whether differences in the BcpB-1 and BcpB-2 POTRA domains account for the proteins’ functional differences, we generated chimeric *bcpB-1* genes that had the regions encoding the POTRA-1 (residues 95–165) or POTRA-1 and POTRA-2 (residues 95–165 and 167–219) domains replaced with the corresponding regions from *bcpB-2* (Fig. 6A). To examine the activity of the BcpB-1 chimeric proteins, they were produced in the *∆bcpB1-4* mutant strain and competed against *∆bcp-1* and *∆bcp-2* recipient bacteria. The chimeric proteins BcpB-1^Chim1^ and BcpB-1^Chim2^ were able to induce a low level of BcpA-1- and BcpA-2-mediated CDI (Fig. 6B and C). Therefore, replacement of the POTRA-1 or POTRA-1 and POTRA-2 domains of BcpB-1 likely allows for increased secretion of BcpA-1 and BcpA-2, although not to the levels observed for full-length BcpB-2.

**Fig 7:**
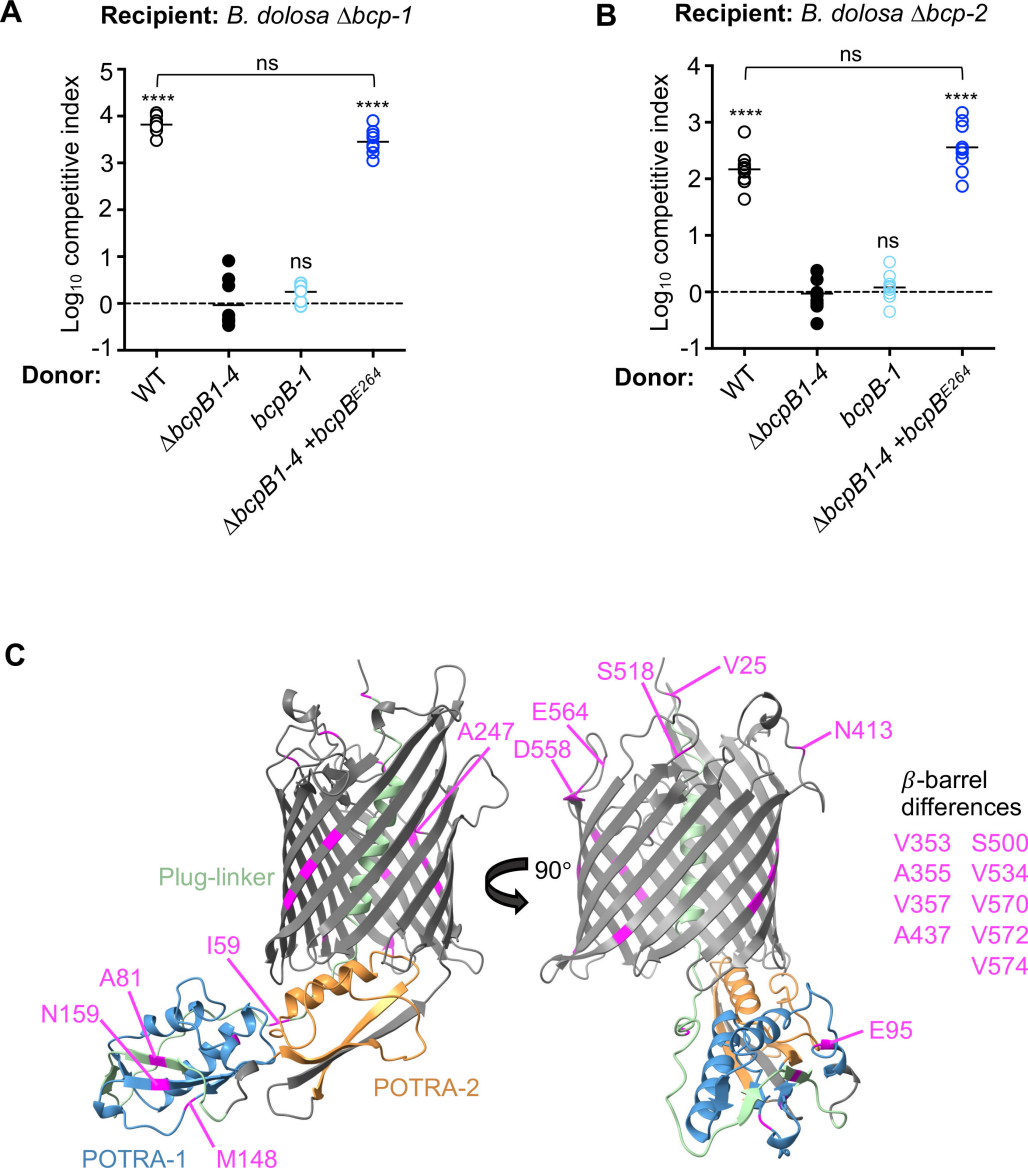
Comparison of *B. thailandensis* BcpB and *B. dolosa* BcpB-1 transporters. (**A and B**) Interbacterial competition assays between the indicated donor cells: *Bd*AU0158 wild type (WT; open circles), ∆*bcpB-1* ∆*bcpB-2* ∆*bcpB-3* ∆*bcpB-4 (∆bcpB1-4;* closed circles), *∆bcpB-2 ∆bcpB-3 ∆bcpB-4* (*bcpB-1*; light blue circles), ∆*bcpB1-4* complemented with overexpressed *Bt*E264 *bcpB* (*∆bcpB1−4 + bcpB^E264^*; dark blue circles), and *Bd*AU0158 (**A**) *∆bcp-1* recipient cells or (**B**) *∆bcp-2* recipient cells. Symbols represent log_10_ CI values from three independent experiments, and horizontal bars show the mean (*n* = 9). Dashed line shows log_10_ competitive index = 0 (no competition). ns, not significant; and ****P < 0.0001; compared to *∆bcpB1-4* donor cells. (**C**) Predicted structure of *B. dolosa* BcpB-1 (without signal sequence) generated by AlphaFold. Plug-linker (green), POTRA-1 (blue), and POTRA-2 (orange) domains are shown. Amino acid residues labeled in pink (numbered according to full-length BcpB-1) represent unique residues that differ in *B.dolosa* BcpB-1 compared to *B. dolosa* BcpB-2 or *B. thailandensis* E264 BcpB protein sequences.

TpsB family proteins like BcpB contain an H1 plug that blocks the barrel pore in the resting conformation; this plug needs to be removed for secretion to occur (15, 16). The plug is connected to the first POTRA domain by a short periplasmic polypeptide, referred to as the linker (Fig. 7C; Fig. S3). It has been shown in other TPS systems that the linker is also necessary for substrate secretion (15, 17, 21, 24). To examine the function of the plug-linker, a third BcpB-1 chimera (BcpB-1^Chim3^) that has the plug and linker regions of BcpB-1 replaced with the regions from BcpB-2 was generated. Donor bacteria producing BcpB-1^Chim3^ were not able to intoxicate either *∆bcp-1* (Fig. 6B) or *∆bcp-2* (Fig. 6C) recipient cells, suggesting that alterations to the plug-linker regions do not explain the low secretion activity for BcpB-1. Lastly, a fourth BcpB-1 chimera (BcpB-1^Chim4^) was generated to replace the plug-linker, POTRA-1, and POTRA-2 domains from BcpB-1 with the corresponding regions from BcpB-2. Similar to BcpB-1^Chim1^ and BcpB-1^Chim2^, donor bacteria producing BcpB-1^Chim4^ showed a ~5-fold and ~10-fold increase in CDI activity as compared to the non-secreting *∆bcpB1-4* mutant.

The three chimeras that contained the BcpB-2 POTRA-1 domain were the only BcpB-1 proteins to show CDI activity. These data suggest that the specificity of the POTRA-1 domain may contribute to the low secretion activity of native BcpB-1. Although we have not ruled out contributions of POTRA-2, only two amino acids differ between the proteins in this region. None of the chimeric BcpB-1 proteins functioned similarly to BcpB-2, indicating that additional differences elsewhere in the protein contribute to the functional differences between BcpB-1 and BcpB-2. Considerable sequence variability between BcpB-1 and BcpB-2 exists C-terminal to the POTRA-2 domain, which is predicted to form the β-barrel (Fig. 7C; Fig. S3).

### BcpB^E264^, a close *B. dolosa* BcpB-1 homolog, can secrete BcpA-1 and BcpA-2

Interestingly, it has been previously reported that the *Burkholderia thailandensis* E264 *bcp* and *B. dolosa* AU0158 *bcp-1* toxin and immunity alleles are functionally interchangeable. The *Bd*AU0158 BcpI-1 and *Bt*E264 BcpI immunity proteins provided cross-protection against both the *Bd*AU0158 BcpA-1 and *Bt*E264 BcpA toxins (6). Even though the *Bd*AU0158 BcpB-1 and *Bt*E264 BcpB proteins are ~95% identical at the amino acid level (Fig. S3), based on our data we hypothesize that they are not functionally identical. Unlike *Bd*AU0158, *Bt*E264 produces only one CDI system with one *bcpB* gene, so we expect the BcpB*^Bt^*^E264^ protein to be functional. To examine the functionality of the *Bt*E264 BcpB protein for secretion of *Bd*AU0158 BcpA proteins, we expressed *Bt*E264 *bcpB* gene in the *Bd*AU0158 ∆*bcpB1-4* mutant. Unlike *Bd*AU0158 BcpB-1, *Bt*E264 BcpB was able to mediate CDI against ∆*bcp-1* (Fig. 7A) or *∆bcp-2* (Fig. 7B) recipient cells at levels not significantly different from wild-type donor cells. These data indicate that the few amino acid differences between the two closely related BcpB proteins are responsible for a large difference in functionality (Fig. S3).

Sequence comparison between *Bd*AU0158 BcpB-1, which appears defective for one or more steps in BcpA secretion or delivery, and the CDI-competent *Bd*AU0158 BcpB-2 and *Bt*E264 BcpB shows a limited number of amino acid differences. Only 20 residues differ in BcpB-1*^Bd^*^AU0158^ as compared to BcpB-2*^Bd^*^AU0158^ or BcpB*^Bt^*^E264^ (Fig. S3). Nine of these residues map to the predicted β-barrel of BcpB-1, including three each in β-strands β7 and β16 (Fig. 7C). Six residues are predicted to be found in or immediately adjacent to extracellular loops. Three residues are predicted in the POTRA-1 domain, and one residue is located in each of the plug-linker region and the region N-terminal to H1.

### Overproduction of BcpB reveals differences in transporter specificity

**Fig 8:**
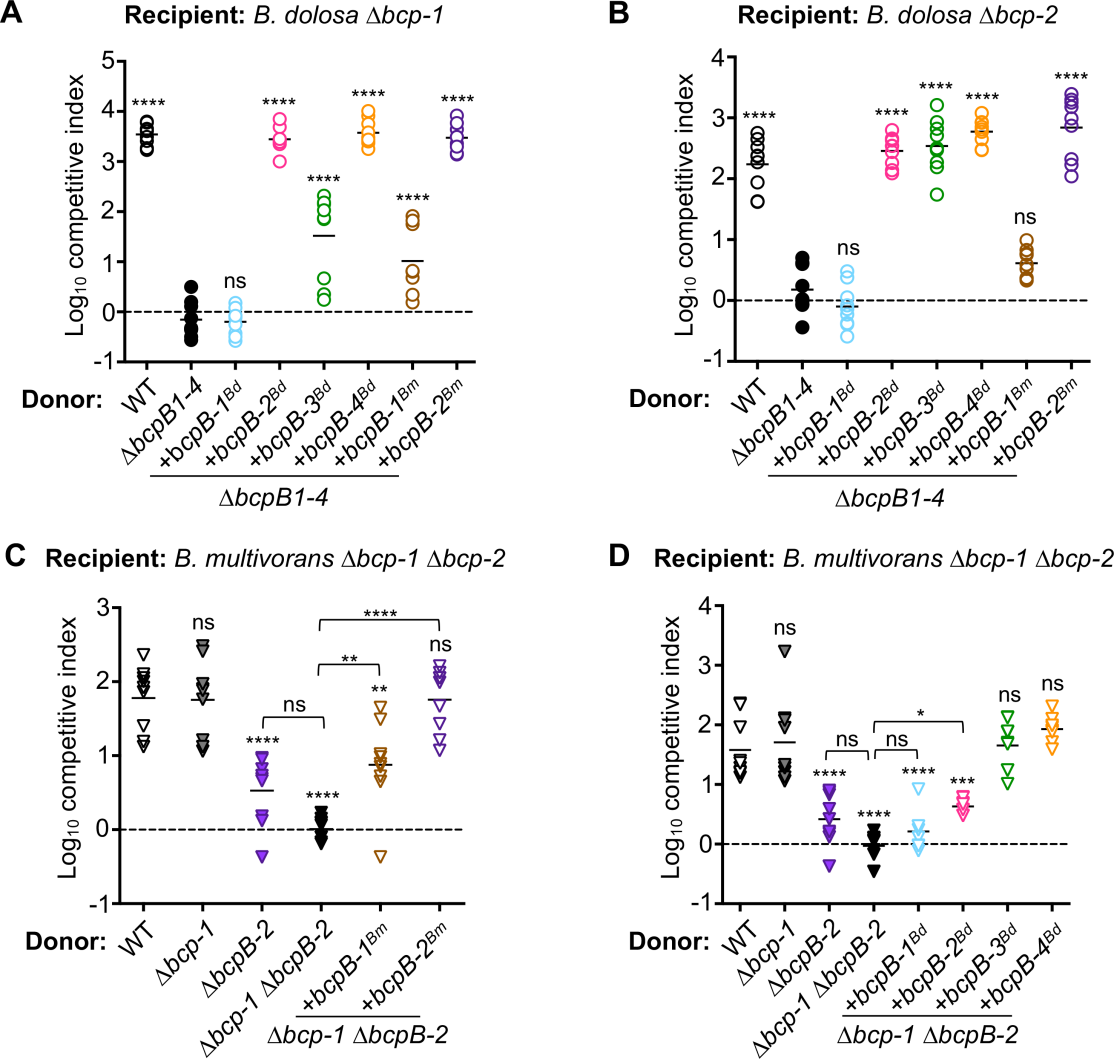
Specificity of *B. dolosa* and *B. multivorans* BcpB transporters for non-cognate BcpA toxins. (**A and B**) Interbacterial competition assays between the indicated donor cells: *Bd*AU0158 wild type (WT; open black circles), ∆*bcpB-1* ∆*bcpB-2* ∆*bcpB-3* ∆*bcpB-4* (*∆bcpB1-4*; closed black circles), or ∆*bcpB1-4* complemented with overexpressed *Bd*AU0158 *bcpB-1* (+*bcpB-1^Bd^*; open blue circles), *bcpB-2* (+*bcpB-2^Bd^*; open pink circles), *bcpB-3* (+*bcpB-3^Bd^*; open green circles), *bcpB-4* (+*bcpB-4^Bd^*; open orange circles), or *Bm*CGD2M *bcpB-1* (+*bcpB-1^Bm^*; open brown circles), *bcpB-2* (*+bcpB-2^Bm^* ; open purple circles), and (**A**) *Bd*AU0158 ∆*bcp-1* recipient cells or (**B**) *Bd*AU0158 ∆*bcp-2* recipient cells. (**C and D**) Interbacterial competition assays between *Bm*CGD2M *∆bcp-1 ∆bcp-2* recipient bacteria and the indicated donor cells: *Bm*CGD2M wild type (open black triangles), ∆*bcp-1* (closed gray triangles), ∆*bcp-2* (closed purple triangles), ∆*bcp1* ∆*bcpB-2* (closed black triangles), and ∆*bcp1* ∆*bcpB-2* complemented with overexpressed (C) *Bm*CGD2M *bcpB-1* (+*bcpB-1^Bm^*; open brown triangles) or *bcpB-2* (+*bcpB-2^Bm^*; open purple triangles) or (**D**) ∆*bcp1* ∆*bcpB-2* complemented with overexpressed *Bd*AU0158 *bcpB-1* (+*bcpB-1^Bd^*; open blue triangles), *bcpB-2* (+*bcpB-2^Bd^*; open pink triangles), *bcpB-3* (+*bcpB-3^Bd^*; open green triangles), or *bcpB-4* (+*bcpB-4^Bd^*; open orange triangles). Symbols represent log_10_ CI values from three independent experiments, and horizontal bars show the mean (*n* = 8 or 9). Dashed line shows log_10_ competitive index = 0 (no competition). ns, not significant; *P < 0.05; **P < 0.01; and ****P < 0.0001; compared to WT donor cells, unless indicated by brackets.

Overexpression of *bcpB-1* did not allow donor bacteria to outcompete ∆*bcp-1* or *∆bcp-2* recipient cells (Fig. 6). To examine whether differences in gene expression could account for other interbacterial toxicity differences observed for the *bcpB* mutant donor cells, we overexpressed the remaining *bcpB* genes in *∆bcpB1-4* donor bacteria. Interestingly, overexpression of *bcpB-2*, *bcpB-3*, or *bcpB-4* allowed intoxication by BcpA-1 (Fig. 8A) or BcpA-2 (Fig. 8B). These data indicate that BcpB-4 can secrete non-cognate toxin when overproduced and suggest that the low activity of natively expressed *bcpB-4* (Fig. 4) is likely due to insufficient gene expression under the conditions used here. These experiments also showed differences in BcpB-3 function. Consistent with the finding that natively expressed *bcpB-3* facilitates reduced BcpA-1-mediated CDI (Fig. 4A), activity of BcpA-1 also appeared diminished here when secretion was dependent on overproduced BcpB-3 (Fig. 8A). By contrast, overproduced BcpB-3 allowed wild-type levels of BcpA-2 toxicity here (Fig. 8B), while competition by donor cells producing native BcpB-3 was reduced (Fig. 4B). These results suggest that BcpB-3 is less competent for BcpA-1 secretion or delivery, while these processes occur more efficiently for BcpA-2.

### BcpB transporter specificity in other *B. cepacia* complex species

Since the *B. dolosa* BcpB transporters appear to have relaxed specificity, we hypothesized that BcpB proteins found in other *Burkholderia* strains may also secrete non-cognate BcpA proteins. *Burkholderia multivorans* CGD2M encodes two CDI systems that can mediate interbacterial toxicity (7), and the associated BcpB proteins are 78%–86% identical to the *Bd*AU0158 transporters (Fig. S1). To assess whether BcpA toxins can be secreted by cross-species BcpB transporters, *Bm*CGD2M BcpB proteins were overproduced in *Bd*AU0158 *∆bcpB1-4* donor bacteria. *Bm*CGD2M BcpB-2 showed wild-type levels of BcpA-1- (Fig. 8A) and BcpA-2 (Fig. 8B)-mediated toxicity, while donors producing *Bm*CGD2M BcpB-1 showed only a low level of BcpA-1 activity. These data suggest that both *Bm*CGD2M BcpB-1 and BcpB-2 can secrete and deliver non-cognate BcpA substrates, although to different extents.

To test whether these *Bm*CGD2M BcpB proteins mediate secretion of non-cognate BcpA proteins in their native organism, we performed interbacterial competition assays with *B. multivorans*. Because previous work from our lab indicated that *Bm*CGD2M CDI system-1 (*bcpAIOB-1*) mediates interbacterial competition only when overexpressed (7), we measured the activity of BcpA-2*^Bm^*^CGD2M^. Reflecting the inactivity of CDI system-1, *∆bcp-1* donor cells outcompeted *∆bcp-1 ∆bcp-2* recipient bacteria at a level similar to wild-type donors (Fig. 8C), indicating that the interbacterial toxicity is due to the BcpA-2 toxin. *Bm*CGD2M donor cells lacking *bcpB-2* outcompeted recipient cells approximately fivefold, but these competitive indices were not significantly different from those of the *bcpB*-deficient *∆bcp-1 ∆bcpB-2* mutant donor cells, possibly reflecting low native expression of *bcpB-1* (Fig. 8C and D). Complementation of the *∆bcp-1 ∆bcpB-2* mutant with overexpressed *Bm*CGD2M *bcpB-1* or *bcpB-2* resulted in interbacterial toxicity, indicating that both transporters can secrete BcpA-2. Only complementation with *bcpB-2* restored interbacterial toxicity to wild-type levels, though, suggesting that BcpA-2 may show preference for its cognate transporter. Together these data indicate that both *B. multivorans* BcpB-1 and BcpB-2 transporters can secrete BcpA-2, but cognate BcpB-2 is necessary for maximum secretion or delivery.

*B. dolosa* BcpB transporters were also capable of secreting *B. multivorans* BcpA-2. Overexpression of *B. dolosa bcpB-3* or *bcpB-4* in the *Bm*CGD2M *∆bcp-1 ∆bcpB-2* donor strain resulted in wild-type levels of CDI (Fig. 8D). *B. multivorans* donor cells producing *B. dolosa* BcpB-2 also inhibited the growth of recipient cells but to a lesser extent than cells producing the native transporter. Consistent with our previous observations, *Bd*AU0158 BcpB-1 did not allow CDI activity in *B. multivorans*.

Together, these findings indicate that the relaxed specificity of the BcpB transporters occurs in several Bcc species that produce multiple CDI systems. By comparing donor cells that overproduced BcpB proteins (Fig. 8), the relative secretion/delivery efficiencies of each BcpA substrate by each transporter could be determined. BcpB-4*^Bd^*^AU0158^ and BcpB-2*^Bm^*^CGD2M^ appeared the most promiscuous, mediating wild-type levels of CDI from all three distinct BcpA substrates (Fig. 8A, B, and D). BcpB-1*^Bm^*^CGD2M^ also secreted all three substrates, but cells producing this transporter displayed diminished interbacterial inhibition (Fig. 8A, B, and C). Interestingly, BcpB-2*^Bd^*^AU0158^ and BcpB-3*^Bd^*^AU0158^ showed variable transporter function. Both proteins mediated wild-type levels of growth inhibition by BcpA-2*^Bd^*^AU0158^ but differed in their abilities to cause CDI by BcpA-1*^Bd^*^AU0158^ and BcpA-2*^Bm^*^CGD2M^ (Fig. 8). Overall, the six distinct BcpB proteins examined here showed variable specificity that depended on the particular BcpA substrate.

## DISCUSSION

**Fig 9:**
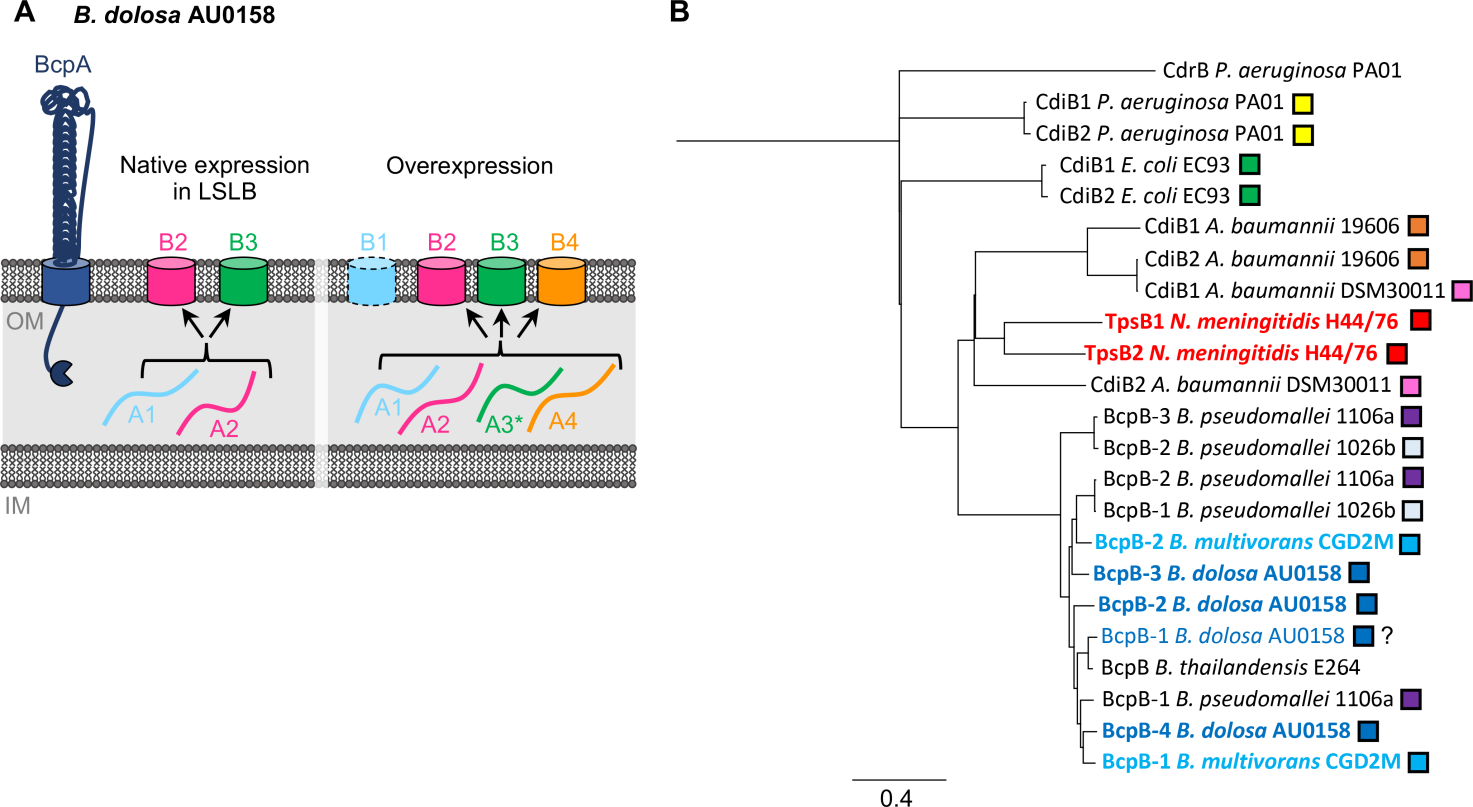
Model for relaxed substrate specificity of BcpB transporters. (**A**) Simplified model of *B. dolosa* BcpA secretion by BcpB transporters. Barrels on cell surface represent BcpB proteins (blue BcpB-1, pink BcpB-2, green BcpB-3, orange BcpB-4), and lines indicate corresponding BcpA proteins (blue BcpA-1, pink BcpA-2, green BcpA-3, orange BcpA-4). Under conditions of native gene expression (left), BcpA-1 and BcpA-2 can be secreted by BcpB-2 or BcpB-3. When *bcpA*/*bcpB* genes are overexpressed (right), all four BcpA toxins can be secreted by BcpB-2, BcpB-3, or BcpB-4. BcpB-1 does not appear to secrete any BcpA protein under the tested conditions. Asterisk denotes that BcpA-3 secretion by BcpB-4 has not been tested. (**B**) Phylogenetic tree based on alignment of CdiB/BcpB proteins and non-CdiB/BcpB TPS protein *P. aeruginosa* CdrB (root). Colored boxes indicate proteins produced within the same strain, and bold colored text indicates transporters for which there is evidence of secretion of cognate and non-cognate substrates. Scale bar represents 0.4 amino acid substitutions per site.

In this study, we investigated the impact of interactions between distinct contact-dependent growth inhibition systems on interbacterial antagonism. Surprisingly, we found that BcpB transporters were dispensable for the secretion of their cognate BcpA toxin. BcpB transporters in multiple *Burkholderia* species showed relaxed specificity and secreted both cognate and non-cognate full-length BcpA toxins. One toxin (*Bd*AU0158 BcpA-1) was secreted exclusively by non-cognate transporters, as its cognate BcpB protein appeared non-functional under the conditions tested here. The promiscuity of the BcpB transporters led to the observation that competition between CDI systems for substrate secretion can occur when transporters are limited. These findings suggest a model in which distinct CDI systems produced by the same organism may not function independently but instead interact to secrete the available pool of toxins (Fig. 9A).

Activity of cognate BcpB/BcpA pairs in *B. dolosa* was sometimes uncoupled, likely due to differences in gene expression. The *bcpA-3* promoter was inactive under laboratory conditions and detection of BcpA-3-mediated CDI required introduction of a strong, constitutive promoter. However, BcpB-3 was highly active under these same conditions and contributed to the secretion of both BcpA-1 and BcpA-2. Expression of *bcpB-3* may be due, in part, to an active promoter upstream of *bcpI-3* that was previously identified (6). Putative promoters driving *bcpA-4* and *bcpB-4* also showed differences in promoter activity. This differential gene expression may produce distinct BcpA/BcpB repertoires in different conditions and, combined with the secretion flexibility we observed, could tune optimal toxin secretion for different environmental niches.

Surprisingly, *B. dolosa* BcpB-1 was not able to secrete or deliver sufficient toxin, either cognate or non-cognate, to mediate interbacterial competition. Changes to the BcpB-1 POTRA-1 domain by replacement with BcpB-2 sequences increased its activity slightly, suggesting that one or more of the 13 amino acid differences in this region contribute to BcpA recognition and/or secretion. In addition, although *Bd*AU0158 BcpB-1 and *Bt*E264 BcpB are ~95% identical, only BcpB*^Bt^*^E264^ appeared functional for BcpA secretion. Comparison of all three transporters (BcpB-1*^Bd^*^AU0158^, BcpB-2*^Bd^*^AU0158^, and BcpB*^Bt^*^E264^) identified 20 residues that are unique to BcpB-1*^Bd^*^AU0158^ (Fig. 7C; Fig. S3). While additional work will be needed to elucidate their potential contributions, three unique residues are located in *β*-barrel strand *β*16, which is part of an interface (*β*1–*β*16) implicated to undergo rearrangements during CdiB secretion (21). It is possible that these sequence differences allow for BcpB-1*^Bd^*^AU0158^ activity under particular environmental conditions. Alternatively, these differences may represent an accumulation of mutations that decreased BcpB-1 function. Unlike *Bt*E264, which produces a single BcpB transporter, detrimental mutations might be tolerated in *B. dolosa* because it produces compensatory BcpB proteins.

The secretion specificity of TpsA-TpsB pairs has been shown to be dependent on recognition of the exoprotein TPS domain (22, 23). Although the BcpA proteins are highly variable, the N-terminus which includes the TPS domain is well conserved (Fig. S2C and S4). *B. dolosa* BcpA-1 and BcpA-2 share only ~37% identity overall, but their TPS domains are 76% identical. By contrast, the TPS domains of *E. coli* and *A. baumannii* CdiA proteins, which are not secreted by each other’s CdiB transporters, share only 46% sequence identity (21). Thus, similarity of the TPS domains among BcpA proteins likely accounts for much of the relaxed specificity observed for the BcpB transporters. However, among the relatively similar *B. cepacia* complex BcpA proteins tested here, there does not appear to be a strong correlation between TPS domain similarity and substrate secretion. For example, the *B. dolosa* BcpA-1 and *B. multivorans* BcpA-2 TPS domains are ~95% identical (Fig. S4), but these substrates utilize BcpB transporters with differing efficiencies (Fig. 8A and D). The amino acid variations between these two TPS domains do not appear to map to a particular region (Fig. S4). However, these results suggest that the closely related BcpA and BcpB proteins examined here may provide a useful framework for investigating additional mechanistic details of CDI system protein secretion and toxin release. Moreover, given the precisely controlled release of partially secreted CdiA/BcpA that has been proposed to occur upon recipient cell engagement (25), it is likely that additional interactions between the substrate protein and BcpB transporter are critical to achieve optimal toxin delivery.

While specificity of a “TpsB” transporter for its partner “TpsA” exoprotein is a hallmark of two-partner secretion systems, substrate flexibility has been observed for other systems. Some organisms encode “orphan” TpsA proteins that do not occur with a partner transporter. *Bordetella bronchiseptica* produces a single transporter, FhaC, which secretes three distinct substrates—FhaB, FhaL, and FhaS (26, 27). Similarly, *Neisseria meningitidis* TpsB2 secretes five TpsA proteins, including cognate, non-cognate, and orphan TpsA proteins, while TpsB1 secretes only two of these (20).

Our results indicate that the relaxed specificity of BcpB transporters leads to interactions between distinct CDI systems produced within the same *B. cepacia* complex strain. Many bacterial species encode two or more complete CDI systems, raising the possibility that similar interactions also occur in these organisms. An examination of >450 clinical and environmental *Burkholderia pseudomallei* isolates showed that 57% harbored two or three distinct *bcpA* (termed “*fhaB3*”) gene clusters (28). *Acinetobacter baumannii*, *A. baylyi*, and 81% of *P. aeruginosa* strains carry two *cdi* loci, several of which have been shown to mediate interbacterial competition (4, 5, 29 - 32). Comparisons of the CdiB/BcpB proteins that co-occur in these species indicate similarities (Fig. 9B), suggesting that CDI system interactions may not only occur in other *Burkholderia* species, such as *B. pseudomallei*, but also in other Gram-negative bacteria that produce multiple CDI systems.

*B. cepacia* complex bacteria can occupy various environmental niches and cause opportunistic infections in immunocompromised individuals. The natural niches in which CDI systems are active or provide a fitness advantage are not known, but it may be advantageous for *Burkholderia* species to produce multiple CDI systems within the same strain. In addition to providing increased toxin diversity and broader immunity, encoding multiple CDI systems may increase secretion efficiency or flexibility by providing additional BcpB transporters.

## MATERIALS AND METHODS

### Bacterial strains and culture conditions

*Burkholderia dolosa* AU0158 (*Bd*AU0158) and *Burkholderia multivorans* CGD2M (*Bm*CGD2M) strains used in this study are listed in Table S1 and were cultured in low salt (0.5% NaCl) Luria-Bertani medium (LSLB). Plasmids were maintained in *Escherichia coli* DH5α and mated into *B. dolosa* or *B. multivorans* using the donor *E. coli* stain RHO3, a 2,6-diaminopimelic acid (DAP) auxotroph (33). For selection of *B. dolosa*, LSLB was supplemented with 250–500 µg/mL kanamycin or 50–125 µg/mL tetracycline. While *B. multivorans* was cultured in LSLB supplemented with 20 µg/mL chloramphenicol, 250 µg/mL kanamycin, or 25–50 µg/mL tetracycline, for selection. *E. coli* strains were cultured in LSLB supplemented, when appropriate, with 100 µg/mL ampicillin, 50 µg/mL kanamycin, 10 µg/mL tetracycline, or 200 µg/mL DAP.

### Genetic manipulations

Plasmids used in this study are listed in Table S2. All plasmid inserts were confirmed by DNA sequencing (Eurofins Genomics or ACGT, Inc.), and bacterial mutant strains were verified by PCR.

In-frame deletion mutations were constructed by allelic exchange using plasmid pEXKm5 (33). Plasmids for gene deletions were constructed by PCR amplification of two fragments: one fragment ~500 bp 5′ to the ORF (including the first three to seven codons) and another ~500 bp 3′ of the ORF (including the last 3–20 codons).

To complement *Bd*AU0158 or *Bm*CGD2M mutants, the genes of interest were PCR amplified and cloned into an *att*Tn7 site delivery plasmid as described in supplemental material. Bacterial mutants were marked with antibiotic resistance cassettes by *att*Tn7 delivery of pUC18Tmini-Tn-Kan (34), pUCTet (10), or for *Bm*CGD2M only, pUCCm (pMA41) (10). Markers were delivered via triparental matings of *E. coli* RHO3 with helper plasmid pTNS3, as previously described (35, 36).

For strains constitutively expressing the *bcp-4* genes, approximately 500 nucleotides 3′ to the *bcpA-4* translational start site were PCR amplified and cloned immediately 3′ to the P_S12_ promoter of plasmid pUCS12. The plasmids were inserted immediately 5′ to the chromosomal copy of each *bcp* locus, the resulting strains were routinely cultured with kanamycin to select for plasmid retention. Additional details of plasmid and strain construction are described in the supplemental material.

### Interbacterial competition assay

Interbacterial competition assays were performed as previously described (36). *B. dolosa* or *B. multivorans* strains carrying antibiotic resistance cassettes at *att*Tn7 sites were cultured overnight without antibiotics and resuspended in sterile PBS to an OD_600_ = 2. Unless noted otherwise, bacteria were mixed at a 1:1 ratio, 20 µL of the mixture was plated on LSLB agar in triplicate, and plates were incubated at 37°C for 24–26 hours. The culture inoculum was plated on LSLB with antibiotic selection to determine the input ratio (donor:recipient) at 0 hours. All donor and recipient bacteria are marked with kanamycin or tetracycline resistance cassettes, respectively. Bacteria were collected from co-cultures with a sterile loop, diluted in sterile PBS, and plated on LSLB with antibiotics to quantify each strain. CI was calculated as a ratio of the donor strain to the recipient strain at 24 hours divided by the input (donor:recipient) ratio at 0 hours. At least three independent experiments were performed in triplicate.

### Beta-galactosidase assay

Reporter strains cultured in LSLB were spotted (20 µL) onto agar plates, incubated overnight at 37°C, and collected in PBS. Cell suspensions were normalized to an OD_600_ = 1.5. Cells were permeabilized with SDS and chloroform, and beta-galactosidase activity was measured as described (6) using a SpectraMax 5M plate reader (Molecular Devices). Three independent experiments were performed, each with three biological replicates.

### Subcellular fractionation, secretion assay, and western blotting

Subcellular fractionation of *Bd*AU0158 was performed as previously described (37) with modifications. Bacterial strains were cultured overnight at 37°C with agitation in LSLB. Cells were harvested by centrifugation at 12,000 × *g* for 15 minutes at 4°C, and the pellet was resuspended to an OD_600_ = 8 in Tris resuspension buffer (50 mM Tris, pH 8 supplemented with Roche Complete Mini EDTA-free Protease Inhibitor Cocktail and Pierce Universal Nuclease for Cell Lysis). Proteins were precipitated from the culture supernatants with 15% trichloroacetic acid, washed with acetone, and dissolved in 10 mM Tris, pH 8 with 2% sodium dodecyl sulfate (SDS) (Tris/SDS buffer). Cells were broken by three passages through a chilled French Pressure cell (40,000 lb/in^2^), and unbroken cells and large debris were removed by two centrifugations at 12,000 × *g* at 4°C for 15 minutes. Total membranes were separated by ultracentrifugation for 15 minutes at 100,000 × *g* at 4°C, and supernatants collected to analyze the cytoplasmic fraction. Total membranes were washed with Tris resuspension buffer, the resulting pellet was resuspended in Tris/SDS buffer. The cytoplasmic fraction was concentrated by methanol-chloroform precipitation, and resulting pellets were suspended in Tris/SDS buffer. Protein concentration for all fractions was determined by Microplate BCA Assay (Pierce).

Equal protein amounts for each fraction were analyzed by SDS-PAGE on Novex 10%–20% Tricine gels (Invitrogen) and transferred to polyvinylidene difluoride 0.2 µM membranes (Invitrogen). Immunoblots were probed as previously described (37) with mouse monoclonal anti-FLAG M2 (Sigma) or anti-*E. coli* RNA Polymerase β (Biolegend) and secondary antibodies coupled to IRDye 800CW (Licor). SYPRO Ruby staining of the gels containing the supernatant and cell lysate was used for visualization of protein loading. Immunoblots and gels were imaged on a Gel Doc EZ Imager (Bio-Rad).

### Bioinformatics and statistics

The putative *bcp-4* locus was identified in the complete genome of *B. dolosa* AU0158 by BLASTp, using *Bd*AU0158 BcpB proteins for queries. Protein alignments were performed using the Clustal W alignment feature of Geneious Prime (2022.2.1), and phylogenetic trees generated using the associated Geneious Tree Builder. Domain predictions were performed using NCBI Conserved Domain search. Predicted *B. dolosa* BcpB-1 structure was generated by AlphaFold (38 - 40), and structure visualized and shaded using UCSF ChimeraX v1.6.1 (41). Data were analyzed by one-way ANOVA with Tukey *post hoc* test or Student’s *t* test using the statistical package in GraphPad Prism (v9).

## Data Availability

The raw data that support the findings of this study are found in the supplemental material (Table S3) or available from the corresponding author upon request.

## SUPPLEMENTAL MATERIAL

Supplemental Material msphere.00303-23-s0001.pdf Supplemental methods, tables, and figures.

ASM does not own the copyrights to Supplemental Material that may be linked to, or accessed through, an article. The authors have granted ASM a non-exclusive, world-wide license to publish the Supplemental Material files. Please contact the corresponding author directly for reuse.
