# Supplementary material for: Relaxed specificity of BcpB transporters mediates interactions between Burkholderia cepacia complex contact-dependent growth inhibition systems
Source: mSphere. 2023 Jul 27;8(4):e00303-23. doi: 10.1128/msphere.00303-23 (PMC10449530; doi:10.1128/msphere.00303-23)
Supplement: Supplemental Material — Supplemental methods, tables, and figures. [file msphere.00303-23-s0001.pdf]

**SUPPLEMENTAL INFORMATION**

# Relaxed specificity of BcpB transporters mediates interactions between *Burkholderia cepacia* complex contact-dependent growth inhibition systems

Zaria K. Elery, Tanya Myers-Morales, Erica D. Phillips, and Erin C. Garcia*

University of Kentucky College of Medicine, Lexington, KY 40536

**SUPPLEMENTAL INFORMATION**

**CONTENT LIST**


**Supplemental Methods**

Genetic manipulations

**Supplemental Figures**

**Fig. S1.** Alignment of *Burkholderia dolosa* and *Burkholderia multivorans* BcpB proteins.

**Fig. S2.** Secretion of BcpA TPS constructs by BcpB transporters.

**Fig. S3.** Examination of *Burkholderia thailandensis* BcpB transporter.

**Fig. S4.** Comparison of *Burkholderia dolosa* and *Burkholderia multivorans* BcpA TPS domains proteins.

**Supplemental Tables**

**Table S1.** *Burkholderia* strains used in this study.

**Table S2.** Plasmids used in this study.

**Table S3.** Raw data ($log_{10}$ competitive index values or Miller units) for all figures.

## Supplemental Methods

### Genetic manipulations

For in-frame deletion of *Bd*AU0158 *bcpB-1,* fragments containing a region of 560bp 5' to the ORF (including the first 3 codons of *bcpB-1*) and 522bp 3' to the ORF (including the last 7 codons) were constructed. For deletion of *Bd*AU0158 *bcpB-2,* fragments containing a region of 552bp 5' to the ORF (including the first 3 codons of *bcpB-2*) and 549bp 3' to the ORF (including the last 6 codons) were constructed. For deletion of *Bd*AU0158 *bcp-4,* fragments containing a region of 446bp 5' to the ORF (including the first 7 codons of *bcpA-4*) and 495bp 3' to the ORF (including the last 20 codons) were constructed. For deletion of *Bm*CGD2M *bcpB-2,* fragments containing a region of 507bp 5' to the ORF (including the first 7 codons of *bcpA-4*) and 505bp 3' to the ORF (including the last 11 codons) were constructed. The fragments for *Bd*AU0158 *bcpB-1*, *bcpB-2, bcp*-4, and *Bm*CGD2M *bcpB-2* were joined by overlap PCR cloned into pExKm5 by restriction digestion, resulting in plasmids pZKE12, pZKE11, pEDP03, and pTMM078, respectively.

For deletion of *BdAU0158 bcpB-3* and *bcpB-4,* deletion fragments were purchased from Thermo Fisher Scientific (GeneArts Strings Gene Synthesis) flanked by restriction sites. For *bcpB-3* the region contained 576bp 5' to the ORF (including the first 3 codons of *bcpB-3*) and 528bp 3' to the ORF (including the last 6 codons of *bcpB-3*). For *bcpB-4* the region contained 238bp 5' to the ORF (including the first 58 codons of *bcpB-4*) and 546bp 3' to the ORF (including the last 6 codons of *bcpB-4*). These fragments were cloned into pExKm5 by restriction digestion, resulting in plasmids pZKE16 and pZKE24, respectively.

To complement mutants, the genes of interest were PCR amplified and cloned into an *att*Tn7 site delivery plasmid. For *Bd*AU0158 *bcpB-1*, *bcpB-2*, *bcpB-3,* and *bcpB-4,* ORFs were cloned by restriction digestion into pUCS12 (1), 3' to the strong, constitutive promoter $P_{S12}$ (*B. thailandensis* E264 *rpsL* gene promoter), resulting in plasmids pZKE13, pZKE14, pZKE15 and pEDP02, respectively. Similarly, *Bm*CGD2M *bcpB-1* and *bcpB-2* were PCR amplified and cloned into pUCS12, resulting in

plasmids pZKE18 and pZKE19, respectively. All genes for complementation were delivered to *att*Tn7 in the *Bd*AU0158 and *Bm*CGD2M genome via triparental mating with helper plasmid pTNS3 as previously described (2,3). To complement the *bcp-4* locus deletion mutant with *bcpI* genes, the *Bd*AU0158 *bcpI-4* and *Bm*CGD2M *bcpI-2* genes were PCR amplified and cloned 3' $P_{S12}$ in pUCTet (1), resulting in plasmids pEDP01 and pECG70, respectively.

For strains constitutively expressing *bcpA-4*, approximately 500 nucleotides 3' to the *bcpA-4* translational start site were PCR amplified and cloned immediately 3' to the $P_{S12}$ promoter of plasmid pUCS12, resulting in plasmid pTMM086. Similarly, a plasmid for constitutive expression of *bcp-3*, pAP29, was obtained from the Cotter laboratory (4). These plasmids were mated into *Bd*AU0158 strains without the pTNS3 helper plasmid (to prevent *att*Tn7 site delivery). Kanamycin-resistant colonies that carried pAP29 or pTMM086 cointegrated 5' to the *bcp-3* or *bcp-4* were obtained and confirmed by PCR, resulting in the positioning of the $P_{S12}$ immediately 5' to the chromosomal copy of each *bcp* locus, similar to previously described strains (3,4). These strains were routinely cultured with kanamycin to select for plasmid retention.

To construct the beta-galactosidase reporters, a ~500bp fragment upstream of *bcpA-1*, *bcpA-2*, *bcpA-4, or bcpB-4* ORF were PCR-amplified and cloned 5' to a promoterless *lacZ* in pUClacZ, resulting in plasmids, pECG99, pECG100, pZKE20, and pZKE21, respectively. The reporter cassettes were delivered to an *att*Tn7 site in the *Bd*AU0158 genome as described above. Control reporters $P_{neg}$-*lacZ* and $P_{S12}$-*lacZ* and previously generated *bcp-3* reporter, pAP24 (4), were also delivered to an *att*Tn7 site in the *Bd*AU0158 genome.

For cloning of the TpsA constructs, nucleotides encoding the N-terminus, including the signal sequence, TPS domain, and part of the FHA repeats, of *bcpA-1* (nucleotides 1-1,602) and *bcpA-2* (nucleotides 1-1,713) were amplified with a 3' primer that added a 1x FLAG tag. Fragments were cloned into pUCS12 via restriction digest resulting in plasmids pZKE22 and pZKE26.

DNA fragments containing chimeric *bcpB-1* of *Bd*AU0158 were ordered from Thermo Fisher Scientific (GeneArts Strings Gene Synthesis). Chimeric gene *bcpB-1*$^{Chim1}$ consists of BdAU0158 *bcpB-1*

coding sequence with nucleotides 283-495 replaced with nucleotides 280-492 from *Bd*AU0158 *bcpB-2*. Chimeric gene *bcpB-1*$^{Chim2}$ consist of *Bd*AU0158 *bcpB-1* coding sequence with nucleotides 283-495 and 499-657 replaced with nucleotides 280-492 and 496-654 from *Bd*AU0158 *bcpB-2*, respectively. Chimeric gene *bcpB-1*$^{Chim3}$ consists of *Bd*AU0158 *bcpB-1* coding sequence with nucleotides 76-207 replaced with nucleotides 94-279 from *Bd*AU0158 *bcpB-2*. Chimeric gene *bcpB-1*$^{Chim4}$ consists of *Bd*AU0158 *bcpB-1* coding sequence with nucleotides 76-207, 283-495, and 499-657 replaced with nucleotides 94-279, 280-492, and 496-654 from *Bd*AU0158 *bcpB-2*, respectively. Restriction digestion was used to clone *bcpB-1*$^{Chim1}$, *bcpB-1*$^{Chim2}$, *bcpB-1*$^{Chim3}$, and *bcpB-1*$^{Chim4}$ into pUCS12 downstream of $P_{S12}$, resulting in plasmids pZKE23, pZKE29, pZKE30, and pZKE31, respectively.

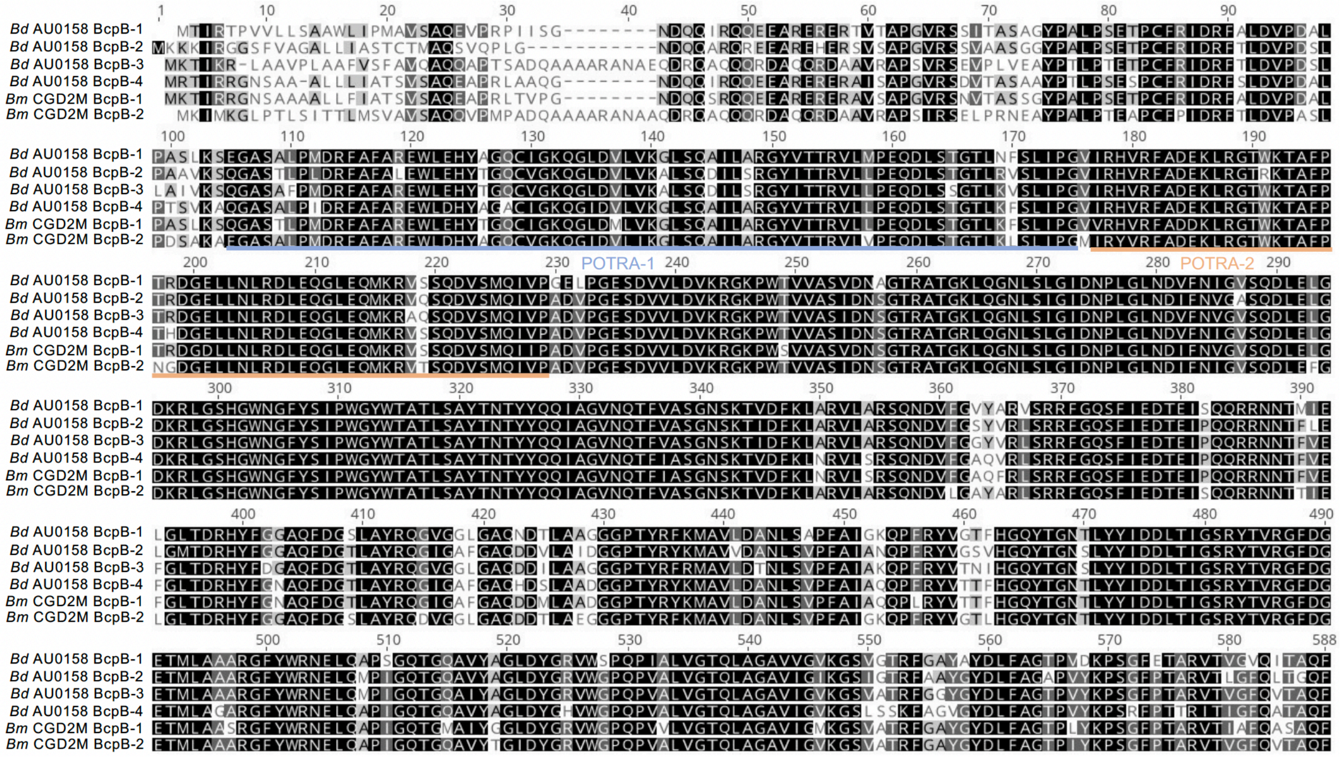


**Supplemental figure 1. Alignment of *Burkholderia dolosa* and *Burkholderia multivorans* BcpB proteins.** Amino acid alignment of *B. dolosa* and *B. multivorans* BcpB proteins. Similarity is denoted by grayscale; residues similar in all sequences are highlighted in black and residues similar in 50% of sequences are highlighted in gray. Regions underlined in blue or orange, represent POTRA-1 or POTRA-2 domains, respectively.

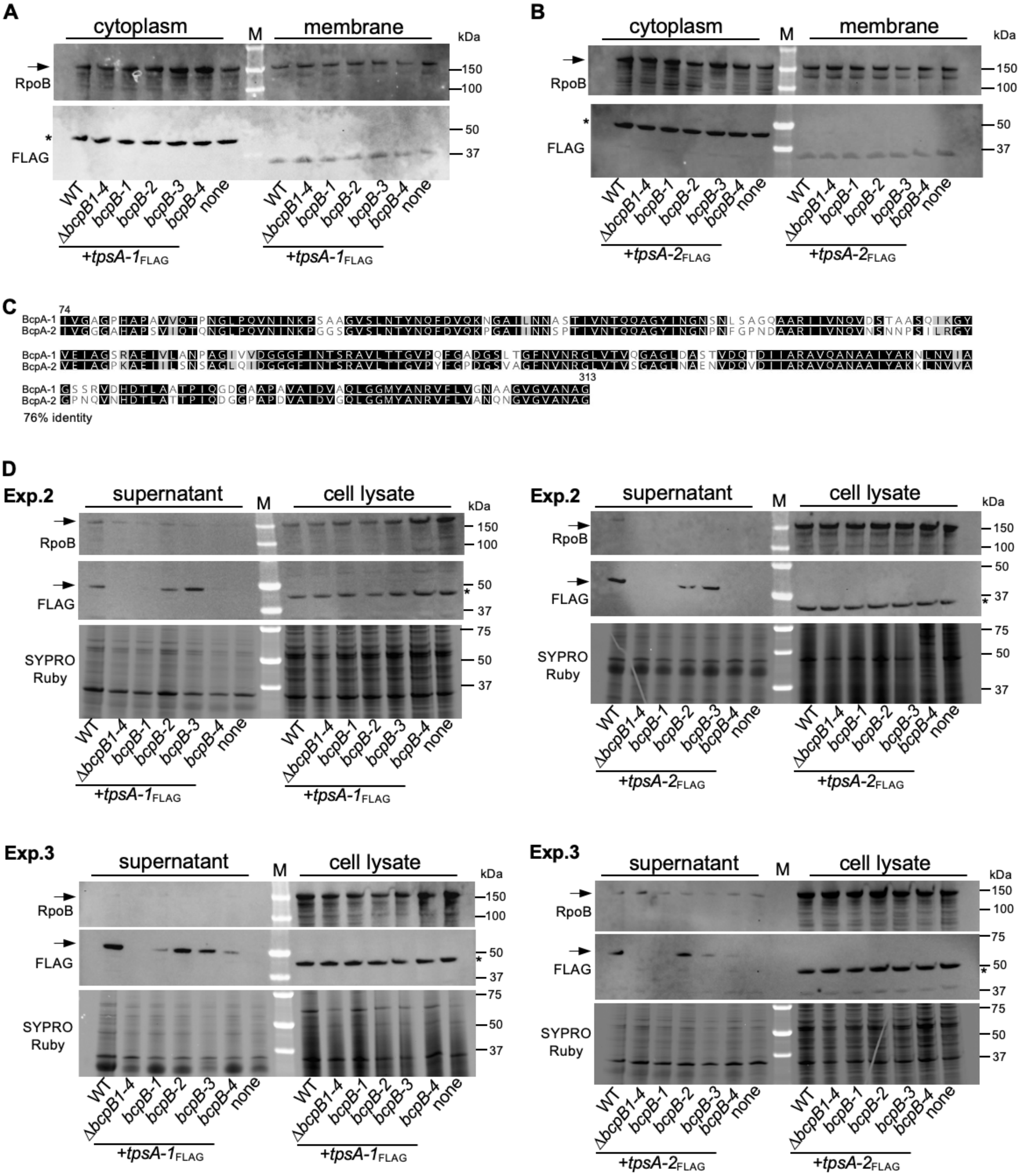
A
cytoplasm
M
membrane
kDa
RpoB
150
100
FLAG
50
37
WT
ΔbcpB1-4
bcpB-1
bcpB-2
bcpB-3
bcpB-4
none
+tpsA-1FLAG
B
cytoplasm
M
membrane
kDa
RpoB
150
100
FLAG
50
37
WT
ΔbcpB1-4
bcpB-1
bcpB-2
bcpB-3
bcpB-4
none
+tpsA-2FLAG
C
74
BcpA-1 IVGAGPHAPAVVQTPNGLPQVNINKPSAAGVSLNTYNQFDVQKNGAILNNASTIVNTQQAGYINGNSNLSAGQAARIIVNQVDSTAASQIKGY
BcpA-2 IVGGGAHAPSVIQTQNGLPQVNINKPGGSGVSLNTYNQFDVQKPGAIINNSPTIVNTQQAGYINGNPNFGPNDAARIIVNQVNSNNPSILRGY
BcpA-1 VEIAGSRAEIVLANPAGIVVDGGGFINTSRAVLTTGVPQFGADGSLTGFNVNRGLVTVQGAGLDASTVDQTDIIARAVQANAAIYAKNLNVIA
BcpA-2 VEIAGPKAEIILSNSAGLQIDGGGFINTSRAVLTTGVPYFGPDGSVAGFNVNRGLVTVSGAGLNAENVDQVDIIARAVQANAAIYAKKLNVVA
313
BcpA-1 GSSRVDHDTLAATPIQGDGAAPAVAIDVAQLGGMYANRVFLVGNAAGVGVANAG
BcpA-2 GPNQVNHDTLATTPIQDGGPAPDVAIDVGQLGGMYANRVFLVANQNGVGVANAG
76% identity
D
Exp.2
supernatant
M
cell lysate
kDa
RpoB
FLAG
SYPRO Ruby
150
100
50
37
75
WT
ΔbcpB1-4
bcpB-1
bcpB-2
bcpB-3
bcpB-4
none
+tpsA-1FLAG
+tpsA-2FLAG
Exp.3
supernatant
M
cell lysate
kDa

**Supplemental figure 2. Secretion of BcpA TPS constructs by BcpB transporters. (A)** Western blots of concentrated culture supernatants and subcellular fractionations of wild-type (WT), Δ*bcpB-1* Δ*bcpB-2* Δ*bcpB-3* Δ*bcpB-4 (*Δ*B1-4*), Δ*bcpB-2* Δ*bcpB-3* Δ*bcpB-4* (*bcpB-1*), Δ*bcpB-1* Δ*bcpB-3* Δ*bcpB-4* (*bcpB-2*), Δ*bcpB-1* Δ*bcpB-2* Δ*bcpB-4* (*bcpB-3*), and Δ*bcpB-1* Δ*bcpB-2* Δ*bcpB-3* (*bcpB-4*) bacteria complemented with either FLAG-tagged BcpA-1 TPS (*tpsA-1)* or **(B)** the FLAG-tagged BcpA-2 TPS (*tpsA-2*). A wild-type (none) strain that lacks a *tpsA* construct was used as a negative FLAG control. Equal protein amounts for each fraction (cytoplasm and total membrane) were resolved on SDS-PAGE gels and blots were probed with anti-FLAG peptide or anti-*E. coli* RNA Polymerase β subunit (RpoB) antibodies. Expected masses for TpsA-1, TpsA-2, and RpoB are ~53, ~56, and 150 kDa, respectively. Arrows show RpoB bands and asterisk indicate nonspecific bands. **(C)** Amino acid alignment of *B. dolosa* BcpA-1 and BcpA-2 TPS domains. Similarity is denoted by grayscale; residues similar in all sequences are highlighted in black and residues similar in 50% of sequences are highlighted in gray. **(D)** Replicates of TPS secretion assays. Western blots of concentrated culture supernatants and whole cell lysate of wild-type (WT), Δ*bcpB-1* Δ*bcpB-2* Δ*bcpB-3* Δ*bcpB-4 (*Δ*B1-4*), Δ*bcpB-2* Δ*bcpB-3* Δ*bcpB-4* (*bcpB-1*), Δ*bcpB-1* Δ*bcpB-3* Δ*bcpB-4* (*bcpB-2*), Δ*bcpB-1* Δ*bcpB-2* Δ*bcpB-4* (*bcpB-3*), and Δ*bcpB-1* Δ*bcpB-2* Δ*bcpB-3* (*bcpB-4*) bacteria complemented with either FLAG-tagged BcpA-1 TPS (*tpsA-1)* or the FLAG-tagged BcpA-2 TPS (*tpsA-2*). A wild-type (none) strain that lacks a *tpsA* construct was used as a negative FLAG control. Equal protein amounts for each fraction (supernatant and cell lysate) were resolved on SDS-PAGE gels. Gels were visualized by SYPRO Ruby staining and blots were probed with anti-FLAG peptide or anti-*E. coli* RNA Polymerase β subunit (RpoB) antibodies. Expected masses for TpsA-1, TpsA-2, and RpoB are ~53, ~56, and 150 kDa, respectively. Arrows show TpsA-FLAG or RpoB bands and asterisks indicate nonspecific bands.

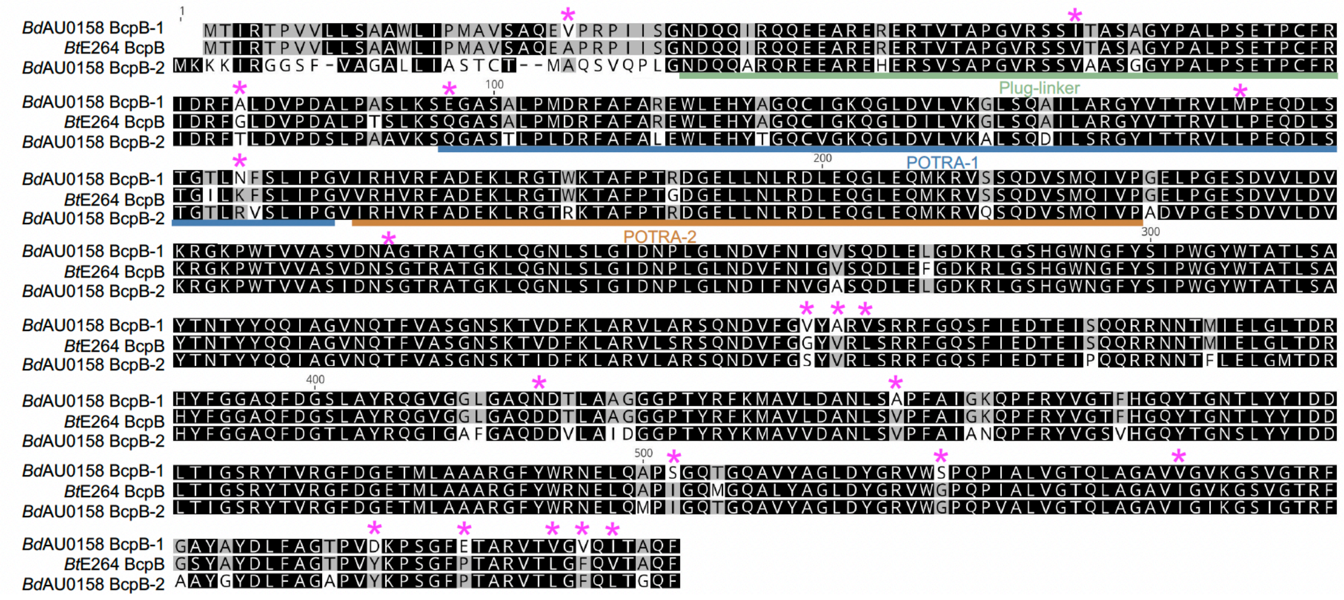

**Supplemental figure 3. Examination of *Burkholderia thailandensis* BcpB transporter. (A)** Amino acid alignment of *Burkholderia dolosa* BcpB-1, BcpB-2 and *Burkholderia thailandensis* BcpB proteins. Similarity is denoted by grayscale; residues similar in all sequences are highlighted in black and residues similar in 50% of sequences are highlighted in gray. Regions underlined in green, blue, or orange, represent the Plug-linker, POTRA-1, or POTRA-2 domains, respectively. Pink asterisks represent unique amino acid residues that differ in BcpB-1 sequence compared to the other two protein sequences.

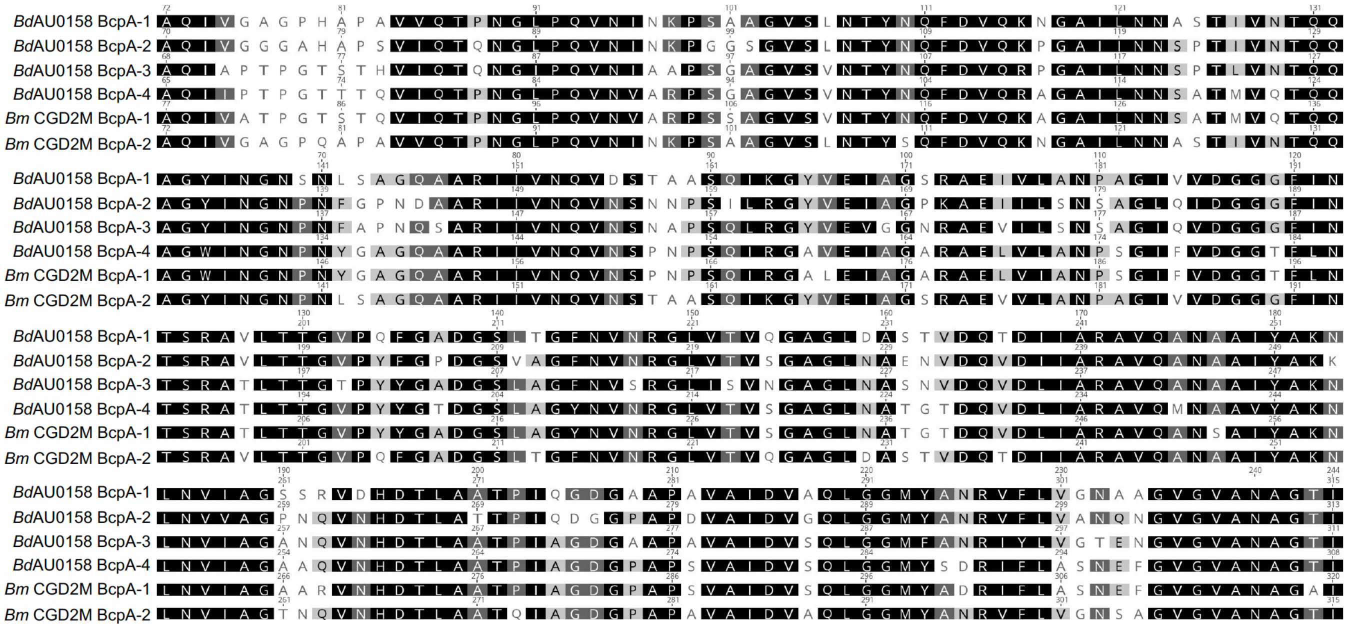

**Supplemental figure 4. Comparison *of B. dolosa* and *B. multivorans* BcpA TPS domains.** Amino acid alignment of *B. dolosa* and *B. multivorans* BcpA TPS domains. Similarity is denoted by grayscale; residues similar in all sequences are highlighted in black and residues similar in 50% of sequences are highlighted in gray.

**Table S1.** ***Burkholderia dolosa*** **and** ***Burkholderia multivorans*** **strains used in this study.**

| Strain | Description | Reference |
|---|---|---|
| *B. dolosa* AU0158 | Wild-type strain | |
| *B. multivorans* CGD2M | Wild-type strain | |
| AU0158 *bcp-3*$^C$ | pAP29 replaces native *bcpA-3* promoter with $P_{S12}$ to constitutively *bcp-3* | (4) |
| AU0158 *bcp-4*$^C$ | pTMM086 replaces native *bcpA-4* promoter with $P_{S12}$ to constitutively *bcp-4* | This study |
| AU0158 Δ*bcp-1* | In-frame deletion of *bcpAIOB-1* | (4) |
| AU0158 Δ*bcp-2* | In-frame deletion of *bcpAIOB-2* | (4) |
| AU0158 Δ*bcp-3* | In-frame deletion of *bcpAIOB-3* | (4) |
| AU0158 Δ*bcp-4* | In-frame deletion of *bcpAIOB-4* | This study |
| AU0158 Δ*bcp-1* Δ*bcp-2* | In-frame deletion of *bcpAIOB-1* and *bcpAIOB-2* | (4) |
| CGD2M Δ*bcp-1* | In-frame deletion of *bcpAIOB-1* in CGD2M | (5) |
| CGD2M Δ*bcp-1* Δ*bcp-2* | In-frame deletion of *bcpAIOB*-1 and *bcpAIB-2* in CGD2M | (5) |
| CGD2M Δ*bcp-1* Δ*bcpB-2* | In-frame deletion of *bcpB-2* in CGD2M *bcpAIOB-1* mutant | This study |
| CGD2M Δ*bcpB-2* | In-frame deletion of *bcpB-2* in CGD2M | This study |
| AU0158 Δ*bcpB-1* | In-frame deletion of Δ*bcpB-1* | This study |
| AU0158 Δ*bcpB-2* | In-frame deletion of Δ*bcpB-2* | This study |
| AU0158 Δ*bcpB-3* | In-frame deletion of Δ*bcpB-3* | This study |

| | | |
|---|---|---|
| AU0158 Δ*bcpB-4* | In-frame deletion of Δ*bcpB-4* | This study |
| AU0158 Δ*bcpB-1* Δ*bcpB-2* Δ*bcpB-3* Δ*bcpB-4 (*Δ*bcpB1-4)* | In-frame deletion of *bcpB-1, bcpB-2, bcpB-3,* and *bcpB-4* | This study |
| AU0158 Δ*bcpB-2* Δ*bcpB-3* Δ*bcpB-4 (bcpB-1)* | In-frame deletion of *bcpB-2, bcpB-3,* and *bcpB-4* | This study |
| AU0158 Δ*bcpB-1* Δ*bcpB-3* Δ*bcpB-4 (bcpB-2)* | In-frame deletion of *bcpB-1, bcpB-3,* and *bcpB-4* | This study |
| AU0158 Δ*bcpB-1* Δ*bcpB-2* Δ*bcpB-4 (bcpB-3)* | In-frame deletion of *bcpB-1, bcpB-2,* and *bcpB-4* | This study |
| AU0158 Δ*bcpB-1* Δ*bcpB-2* Δ*bcpB-3 (bcpB-4)* | In-frame deletion of *bcpB-1, bcpB-2,* and *bcpB-3* | This study |
| AU0158 Δ*bcpB-1* Δ*bcpB-2* | In-frame deletion of *bcpB-1* and *bcpB-2* | This study |
| AU0158 Δ*bcpB-1* Δ*bcpB-2* *bcp-1*$^{C}$ | Δ*bcpB-1* Δ*bcpB-2::*pS12AP6 replaces native *bcpA-1* promoter with $P_{S12}$ to constitutively *bcp-1* | This study |
| AU0158 Δ*bcpB-1* Δ*bcpB-2* *bcp-2*$^{C}$ | Δ*bcpB-1* Δ*bcpB-2*::pS12AP7 replaces native *bcpA-2* promoter with $P_{S12}$ to constitutively *bcp-2* | This study |
| AU0158 Δ*bcp-1* Δ*bcpB-2* | In-frame deletion of *bcpB-2* in Δ*bcp-1* mutant | This study |
| AU0158 Δ*bcp-2* Δ*bcpB-1* | In-frame deletion of *bcpB-1* in Δ*bcp-2* mutant | This study |

**Table S2. Plasmids used in this study.**

| Plasmids | Backbone | Description | Antibiotic Resistance | Reference |
|---|---|---|---|---|
| pEXKm5 | | Allelic exchange vector | Kan | (6) |
| pUC18Tmini-Tn7T-Km | | To deliver Kan resistance cassette to *att*Tn7 site | Amp, Kan | (2) |
| pUC18Tmini-Tn7T- Tet | pUC18Tmini-Tn7T-Km | To deliver Tet resistance cassette to *att*Tn7 site | Amp, Tet | (1) |
| pTNS3 | | Helper plasmid to deliver cassettes to *att*Tn7 | Amp | (7) |
| pUCS12km | pUC18Tmini-Tn7T-Km | To deliver $P_{S12}$-driving cassettes to *att*Tn7 site | Amp, Kan | (1) |
| pECG103 | pUC18Tmini-Tn7T-Tet | To deliver constitutively-expressed ($P_{S12}$) *bcpI-1* to *att*Tn7 site | Amp, Tet | This study |
| pECG104 | pUC18Tmini-Tn7T-Tet | To deliver constitutively-expressed ($P_{S12}$) *bcpI-2* to *att*Tn7 site | Amp, Tet | This study |
| pAP42 | pUC18Tmini-Tn7T-Tet | To deliver constitutively expressed ($P_{S12}$) *bcpI-3* to *att*Tn7 site | Amp, Tet | (4) |
| pEDP01 | pUC18Tmini-Tn7T-Tet | To deliver constitutively expressed ($P_{S12}$) *bcpI-4* to *att*Tn7 site | Amp, Tet | This study |
| pECG70 | pUC18Tmini-Tn7T-Tet | To deliver *Bm*CGD2M constitutively expressed ($P_{S12}$) *bcpI-2* to *att*Tn7 site | Amp, Tet | (5) |
| pAP29 | pUC18Tmini-Tn7T-Km | 1st 501bp of *Bd*AU0158 *bcpA-3* used to generate *Bd*AU0158 *bcp-3*$^{C}$ | Amp, Kan | (4) |
| pTMM086 | pUC18Tmini-Tn7T-Km | 1st 500bp of *Bd*AU0158 *bcpA-4* used to generate *Bd*AU0158 *bcp-4*$^{C}$ | Amp, Kan | This study |
| pZKE11 | pEXKm5 | To generate in-frame deletion of *bcpB-2* | Kan | This study |

| | | | | |
|---|---|---|---|---|
| pZKE12 | pEXKm5 | To generate in-frame deletion of *bcpB-1* | Kan | This study |
| pZKE16 | pEXKm5 | To generate in-frame deletion of *bcpB-3* | Kan | This study |
| pZKE24 | pEXKm5 | To generate in-frame deletion of *bcpB-4* | Kan | This study |
| pZKE13 | pUC18Tmini-Tn7T-Km | To deliver constitutively expressed ($P_{S12}$) *bcpB-1* to *att*Tn7 site | Amp, Kan | This study |
| pZKE14 | pUC18Tmini-Tn7T-Km | To deliver constitutively expressed ($P_{S12}$) *bcpB-2* to *att*Tn7 site | Amp, Kan | This study |
| pZKE15 | pUC18Tmini-Tn7T-Km | To deliver constitutively expressed ($P_{S12}$) *bcpB-3* to *att*Tn7 site | Amp, Kan | This study |
| pEDP02 | pUC18Tmini-Tn7T-Km | To deliver constitutively expressed ($P_{S12}$) *bcpB-4* to *att*Tn7 site | Amp, Kan | This study |
| pTMM078 | pEXKm5 | To generate in-frame deletion of *Bm*CGD2M *bcpB-2* | Kan | This study |
| pZKE18 | pUC18Tmini-Tn7T-Km | To deliver *Bm*CGD2M constitutively expressed ($P_{S12}$) *bcpB-1* to *att*Tn7 site | Amp, Kan | This study |
| pZKE19 | pUC18Tmini-Tn7T-Km | To deliver *Bm*CGD2M constitutively expressed ($P_{S12}$) *bcpB-2* to *att*Tn7 site | Amp, Kan | This study |
| pZKE22 | pUC18Tmini-Tn7T-Km | To deliver constitutively expressed ($P_{S12}$) TPS domain of *bcpA-1* to *att*Tn7 site | Amp, Kan | This study |
| pZKE26 | pUC18Tmini-Tn7T-Km | To deliver constitutively expressed ($P_{S12}$) TPS domain of *bcpA-2* to *att*Tn7 site | Amp, Kan | This study |
| pZKE25 | pUC18Tmini-Tn7T-Tet | To deliver constitutively-expressed ($P_{S12)}$ *bcpI-4* to *att*Tn7 site | Amp, Tet | This study |
| pEDP04 | pEXKm5 | To generate in-frame deletion of *bcp-4* | Kan | This study |
| pZKE23 | pUC18Tmini-Tn7T-Km | To deliver constitutively expressed ($P_{S12}$) *bcpB-1* chimera 1 to *att*Tn7 site | Amp, Kan | This study |

| | | | | |
|---|---|---|---|---|
| pZKE29 | pUC18Tmini-Tn7T-Km | To deliver constitutively expressed ($P_{S12}$) *bcpB-1* chimera 2 to *att*Tn7 site | Amp, Kan | This study |
| pZKE30 | pUC18Tmini-Tn7T-Km | To deliver constitutively expressed ($P_{S12}$) *bcpB-1* chimera 3 to *att*Tn7 site | Amp, Kan | This study |
| pZKE31 | pUC18Tmini-Tn7T-Km | To deliver constitutively expressed ($P_{S12}$) *bcpB-1* chimera 4 to *att*Tn7 site | Amp, Kan | This study |
| pS12AP6 | pUC18Tmini-Tn7T-Km | To integrate $P_{S12}$ promoter 5' to *bcp-1* | Kan | (3) |
| pS12AP7 | pUC18Tmini-Tn7T-Km | To integrate $P_{S12}$ promoter 5' to *bcp-2* | Kan | (3) |
| pECG10 | pUC18Tmini-Tn7T-Km | To deliver $P_{S12}$-*lacZ* to *att*Tn7 site | Amp, Kan | (1) |
| pUClacZ | pUC18Tmini-Tn7T-Km | To deliver $P_{neg}$-*lacZ* (promoterless) to *att*Tn7 site | Amp, Kan | (1) |
| $P_{bcp-1}$-*lacZ* | pUC18Tmini-Tn7T-Km | To deliver $P_{bcpA-1}$-*lacZ* to *att*Tn7 site | Amp, Kan | (4) |
| $P_{bcp-2}$-*lacZ* | pUC18Tmini-Tn7T-Km | To deliver $P_{bcpA-2}$-*lacZ* to *att*Tn7 site | Amp, Kan | (4) |
| $P_{bcp-3}$-*lacZ* | pUC18Tmini-Tn7T-Km | To deliver $P_{bcpA-3}$-*lacZ* to *att*Tn7 site | Amp, Kan | (4) |
| pZKE20 | pUC18Tmini-Tn7T-Km | To deliver $P_{bcpA-4}$-*lacZ* to *att*Tn7 site | Amp, Kan | This study |
| pZKE21 | pUC18Tmini-Tn7T-Km | To deliver $P_{bcpB-4}$-*lacZ* to *att*Tn7 site | Amp, Kan | This study |

Kan, Kanamycin; Amp, Ampicillin; Tet, Tetracycline

Raw data (log10 competitive index values or Miller units) for all figures

**Figure**

1B

| | ***PbcpA-1-lacZ*** | **P*bcpA-2-lacZ*** | **P*bcpA-3-lacZ*** | **P*bcpA-4-lacZ*** | **P*bcpB-4-lacZ*** | ***PS12-lacZ*** | **Pneg-lacZ** |
|---|---|---|---|---|---|---|---|
| Exp 1 | 323.28 | 219.28 | 18.70 | 346.82 | 50.10 | 0.00 | 12723.45 |
| | 258.50 | 175.68 | 10.83 | 389.07 | 40.88 | 0.00 | 13493.12 |
| | 248.59 | 149.63 | 21.46 | 403.35 | 18.73 | 0.00 | 12355.39 |
| Exp 2 | 282.29 | 205.37 | 0.00 | 355.96 | 13.52 | 0.00 | 12779.30 |
| | 282.54 | 224.05 | 0.00 | 375.81 | 17.07 | 0.00 | 12450.81 |
| | 292.03 | 210.44 | 0.00 | 342.41 | 0.00 | 0.00 | 12339.48 |
| Exp 3 | 209.30 | 144.98 | 0.00 | 286.80 | | 0.00 | 9931.64 |
| | 187.95 | 140.21 | 0.00 | 283.93 | | 0.00 | 9752.95 |
| | 207.01 | 150.04 | 0.00 | 279.48 | | 0.00 | 9515.06 |
| Exp 4 | 164.59 | 115.20 | 0.00 | 244.88 | 2.84 | 0.00 | 9404.45 |
| | 169.49 | 111.11 | 0.00 | 240.93 | 2.60 | 0.00 | 9009.37 |
| | 152.45 | 118.93 | 0.00 | 250.65 | 15.54 | 0.00 | 8925.58 |

1C

| **Donor** | **WT** | **WT** | **WT** | ***bcp-4***$^{C}$ | ***bcp-4***$^{C}$ | ***bcp-4***$^{C}$ | **WT** | **WT** | **WT** | ***bcp-4***$^{C}$ | ***bcp-4***$^{C}$ | ***bcp-4***$^{C}$ |
|---|---|---|---|---|---|---|---|---|---|---|---|---|
| **Recipient** | ***∆bcp-4*** | ***bcpI-4*** | ***bcpI-2***$^{Bm}$ | ***∆bcp-4*** | ***bcpI-4*** | ***bcpI-2***$^{Bm}$ | ***∆bcp-4*** | ***bcpI-4*** | ***bcpI-2***$^{Bm}$ | ***∆bcp-4*** | ***bcpI-4*** | ***bcpI-2***$^{Bm}$ |
| Exp 1 | 0.53 | 0.39 | 0.67 | 0.92 | -0.11 | 0.95 | 0.72 | 0.35 | 0.55 | 1.65 | -0.18 | 1.64 |
| | 0.61 | 0.49 | 0.51 | 1.40 | -0.03 | 0.78 | 0.52 | 0.65 | 0.33 | 2.01 | -0.15 | 1.85 |
| | 0.57 | 0.73 | 0.56 | 0.99 | -0.15 | 1.11 | 0.37 | 0.59 | 0.40 | 2.03 | -0.16 | 2.19 |
| Exp 2 | 1.01 | 0.62 | 0.62 | 1.22 | -0.57 | 1.18 | 0.66 | 0.25 | 0.93 | 2.43 | -0.22 | 1.59 |
| | 1.09 | 1.02 | 1.03 | 1.20 | 0.08 | 1.21 | 0.88 | 0.47 | 0.93 | 1.93 | -0.24 | 1.68 |
| | 1.00 | 1.01 | 1.10 | 1.12 | -0.17 | 1.38 | 0.64 | 0.98 | 0.93 | 1.82 | -0.09 | 1.64 |
| Exp 3 | 0.50 | 0.35 | 0.57 | 0.61 | -0.20 | 1.09 | 0.59 | 0.60 | 0.82 | 1.55 | -0.17 | 1.81 |
| | 0.63 | 0.53 | 0.76 | 0.61 | 0.02 | 0.67 | 0.70 | 0.43 | 0.99 | 1.61 | 0.14 | 1.68 |
| | 0.51 | 0.63 | 0.53 | 0.78 | -0.05 | 1.00 | 0.57 | 0.96 | 0.60 | 1.71 | -0.02 | 1.32 |

2A

| **Donor** | **WT** | ***ΔbcpB-1*** | ***+bcpB-1*** | **WT** |
|---|---|---|---|---|

| Recipient | Δ*bcp-1* | Δ*bcp-1* | Δ*bcp-1* | immunity |
|---|---|---|---|---|
| Exp 1 | 2.46 | 2.38 | 2.56 | 0.64 |
| | 2.46 | 2.52 | 2.33 | 0.63 |
| | 2.51 | 2.38 | 2.26 | 0.53 |
| Exp 2 | 3.23 | 3.21 | 3.13 | 0.58 |
| | 2.96 | 2.92 | 3.17 | 0.23 |
| | 3.07 | 3.10 | 3.00 | 0.24 |
| Exp 3 | 2.87 | 2.48 | 2.82 | 0.24 |
| | 3.08 | 2.51 | 2.49 | 0.07 |
| | 2.73 | 2.67 | 2.35 | -0.01 |

2B

| Donor | WT | Δ*bcpB-2* | +*bcpB-2* | WT |
|---|---|---|---|---|
| Recipient | Δ*bcp-2* | Δ*bcp-2* | Δ*bcp-2* | immunity |
| Exp 1 | 2.5 | 1.84 | 2.76 | 0.37 |
| | 2.46 | 1.86 | 2.79 | 0.12 |
| | 2.55 | 1.93 | 2.71 | 0.52 |
| Exp 2 | 2.74 | 1.74 | 3.2 | 0.46 |
| | 2.84 | 1.69 | 3.05 | -0.09 |
| | 2.7 | 1.54 | 2.59 | 0.57 |
| Exp 3 | 2.67 | 1.6 | 2.82 | 0.02 |
| | 2.67 | 1.58 | 2.56 | 0.15 |
| | 2.98 | 2.07 | 2.46 | 0.12 |

2C

| Donor | *bcp-3*$^{C}$ | Δ*bcpB-3* | +*bcpB-3* | *bcp-3*$^{C}$ |
|---|---|---|---|---|
| Recipient | Δ*bcp-3* | Δ*bcp-3* | Δ*bcp-3* | immunity |
| Exp 1 | 2.95 | 2.09 | 4.31 | 0.00 |
| | 4.10 | 2.05 | 5.61 | -0.19 |
| | 5.20 | 2.21 | 4.18 | -0.21 |

| | | | | |
|---|---|---|---|---|
| Exp 2 | 4.97 | 1.82 | 5.73 | 0.15 |
| | 5.30 | 1.67 | 5.66 | -0.10 |
| | 5.36 | 1.87 | 5.33 | 0.01 |
| Exp 3 | 4.17 | 1.62 | 6.27 | -0.27 |
| | 4.71 | 1.59 | 4.6 | -0.42 |
| | 5.56 | 1.55 | 5.11 | -0.26 |

2D

| **Donor** | ***bcp-4*$^C$** | ***ΔbcpB-4*** | ***+bcpB-4*** | ***bcp-4*$^C$** | ***+bcpB-4*** |
|---|---|---|---|---|---|
| **Recipient** | **Δ*bcp-4*** | **Δ*bcp-4*** | **Δ*bcp-4*** | **immunity** | **immunity** |
| Exp 1 | 1.45 | 1.60 | 4.58 | -0.44 | |
| | 1.35 | 1.31 | 3.68 | -0.57 | |
| | 1.45 | 1.35 | 4.98 | -0.49 | |
| Exp 2 | 1.32 | 1.54 | 3.72 | -0.43 | |
| | 1.41 | 1.40 | 5.35 | 0.38 | |
| | 1.22 | 1.63 | 5.92 | 0.29 | |
| Exp 3 | 1.64 | 1.40 | 5.34 | -0.19 | |
| | 1.64 | 1.20 | 5.33 | 0.05 | |
| | 1.45 | 1.54 | 5.73 | -0.07 | |
| Exp 4 | 1.85 | | 5.19 | -0.85 | -0.41 |
| | 1.63 | | 5.37 | -0.73 | -0.04 |
| | 1.43 | | 4.99 | -0.53 | -0.18 |
| Exp 5 | 2.1 | | 5.43 | 0.15 | 0.09 |
| | 2.04 | | 5.53 | -0.18 | 0.3 |
| | 1.96 | | 5.51 | -0.15 | 0.13 |
| Exp 6 | 2.29 | | 5.42 | -0.06 | 0.16 |
| | 2.8 | | 5.25 | 0.08 | 0.15 |
| | 1.49 | | 5.24 | -0.19 | 0.29 |

4A

| Donor / Recipient | WT / Δ*bcp-1* | Δ*bcp1-4* / Δ*bcp-1* | *bcpB-1* / Δ*bcp-1* | *bcpB-2* / Δ*bcp-1* | *bcpB-3* / Δ*bcp-1* | *bcpB-4* / Δ*bcp-1* | WT / immunity |
|---|---|---|---|---|---|---|---|
| Exp 1 | 4.07** | 0.52** | 0.42** | 2.19 | 1.63 | 0.57 | 0.95 |
| | 3.78** | 0.38** | 0.35** | 2.52 | 1.39 | 0.63 | 0.96 |
| | 4.02** | 0.91** | 0.37** | 2.53 | 1.60 | 0.63 | 0.96 |
| Exp 2 | 3.90** | -0.38** | 0.44** | 2.11 | 1.19 | 0.25 | 0.71 |
| | 3.48** | -0.33** | 0.37** | 2.30 | 0.83 | 0.17 | 0.76 |
| | 3.78** | -0.47** | -0.06** | 2.13 | 0.79 | -0.12 | 0.63 |
| Exp 3 | 3.85** | -0.25** | 0.04** | 1.98 | 1.05 | -0.06 | 0.65 |
| | 3.70** | -0.34** | 0.04** | 2.50 | 1.30 | -0.06 | 0.64 |
| | 3.78** | -0.37** | 0.26** | 2.05 | 0.74 | -0.17 | 0.79 |

** Values are the same as in 7A (first, second, and third columns) as both experiments (4A and 7A) were performed at the same time using the same controls.

4B

| Donor / Recipient | WT / Δ*bcp-2* | Δ*bcp1-4* / Δ*bcp-2* | *bcpB-1* / Δ*bcp-2* | *bcpB-2* / Δ*bcp-2* | *bcpB-3* / Δ*bcp-2* | *bcpB-4* / Δ*bcp-2* | WT / immunity |
|---|---|---|---|---|---|---|---|
| Exp 1 | 2.12** | -0.56** | 0.08** | 1.96 | 1.69 | 0.38 | 0.35 |
| | 2.00** | 0.38** | 0.14** | 3.09 | 1.59 | 0.42 | 0.63 |
| | 2.83** | -0.20** | 0.53** | 2.42 | 1.67 | 0.44 | 0.60 |
| Exp 2 | 2.25** | -0.15** | 0.04** | 2.48 | 1.37 | 0.24 | 1.05 |
| | 2.18** | -0.01** | 0.28** | 1.87 | 1.23 | 0.01 | 0.84 |
| | 2.20** | 0.37** | -0.08** | 2.08 | 1.25 | 0.27 | 0.80 |
| Exp 3 | 2.33** | -0.06** | -0.02** | 1.91 | 0.96 | 0.41 | 0.48 |
| | 1.95** | 0.22** | 0.10** | 1.45 | 1.33 | -0.45 | 0.89 |
| | 1.64** | -0.25** | -0.35** | 1.85 | 1.30 | 0.31 | 0.68 |

** Values are the same as in 7B (first, second, and third columns) as both experiments (4B and 7B) were performed at the same time using the same controls.

4C

| Donor | *bcp-3*$^{C}$ | Δ*bcp1-4* | *bcpB-1* | *bcpB-2* | *bcpB-3* | *bcpB-4* | *bcp-3*$^{C}$ |
|---|---|---|---|---|---|---|---|
| **Recipient** | Δ*bcp-3* | Δ*bcp-3* | Δ*bcp-3* | Δ*bcp-3* | Δ*bcp-3* | Δ*bcp-3* | **immunity** |
| Exp 1 | 5.21 | 0.80 | 0.20 | 2.46 | 5.53 | 0.28 | -0.21 |
| | 4.45 | 0.79 | 0.10 | 2.59 | 5.63 | 0.03 | -0.25 |
| | 5.08 | -0.60 | 0.15 | 2.51 | 5.10 | -0.06 | -0.25 |
| Exp 2 | 5.18 | -0.54 | 0.43 | 2.30 | 5.47 | 0.06 | -0.36 |
| | 5.36 | -0.34 | 0.34 | 2.54 | 5.56 | 0.51 | -0.36 |
| | 5.41 | -0.15 | 0.46 | 2.44 | 6.08 | -0.09 | -0.57 |
| Exp 3 | 2.97 | -0.45 | 0.52 | 2.64 | 2.93 | 0.07 | -0.04 |
| | 2.59 | 0.14 | 0.51 | 2.68 | 2.71 | 0.04 | -0.51 |
| | 2.58 | -0.13 | 0.31 | 2.62 | 2.89 | 0.05 | -0.48 |

4D

| Donor | *bcp-4*$^{C}$ | Δ*bcp1-4* | *bcpB-1* | *bcpB-2* | *bcpB-3* | *bcpB-4* | *bcp-4*$^{C}$ |
|---|---|---|---|---|---|---|---|
| **Recipient** | Δ*bcp-4* | Δ*bcp-4* | Δ*bcp-4* | Δ*bcp-4* | Δ*bcp-4* | Δ*bcp-4* | **immunity** |
| Exp 1 | 1.44 | 0.00 | 0.20 | 0.53 | 0.56 | 0.00 | 0.33 |
| | 1.65 | -0.04 | 0.17 | 0.58 | 0.72 | -0.22 | 0.33 |
| | 1.82 | -0.23 | 0.10 | 0.85 | 0.72 | 0.07 | 0.28 |
| Exp 2 | 2.82 | -0.51 | 0.70 | 1.54 | 0.64 | 0.00 | -0.36 |
| | 2.89 | -0.13 | 0.68 | 1.13 | 0.72 | 0.26 | 0.09 |
| | 2.26 | -0.29 | 0.58 | 1.57 | 0.42 | 0.23 | -0.37 |
| Exp 3 | 1.70 | -0.32 | 0.49 | 2.44 | 0.65 | -0.19 | -0.21 |
| | 1.77 | -0.56 | 0.48 | 2.13 | 1.35 | -0.06 | -0.47 |
| | 2.09 | -0.07 | 0.24 | 2.15 | 0.81 | -0.03 | -0.27 |

5A

| Donor | **WT** | Δ*bcpB1-2* | Δ*bcpB1-3* | *bcp-2*$^{C}$ Δ*bcpB1-2* | Δ*bcp-2* Δ*bcpB-1* | Δ*bcpB1-2* |
|---|---|---|---|---|---|---|
| **Recipient** | Δ*bcp-1* | Δ*bcp-1* | Δ*bcp-1* | Δ*bcp-1* | Δ*bcp-1* Δ*bcp-2* | **immunity** |

| | | | | | | |
|---|---|---|---|---|---|---|
| Exp 1 | 3.99 | 1.59 | 0.12 | -0.09 | 2.76 | -0.62 |
| | 3.54 | 1.21 | 0.12 | -0.03 | 2.54 | -0.66 |
| | 3.01 | 1.43 | 0.17 | -0.91 | 2.55 | -0.85 |
| Exp 2 | 3.78 | 1.34 | -0.20 | 0.37 | 3.19 | -0.26 |
| | 3.28 | 1.43 | 0.11 | -0.04 | 2.94 | -0.59 |
| | 3.84 | 1.19 | 0.44 | 0.25 | 3.10 | -0.51 |
| Exp 3 | 3.98 | 1.50 | -0.18 | 0.03 | 2.69 | -0.37 |
| | 3.68 | 1.41 | -0.03 | 0.11 | 2.52 | -0.52 |
| | 3.83 | 1.36 | -0.49 | 0.04 | 2.02 | -1.03 |

6B

| **Donor** | **WT** | ***Δbcp1-4*** | ***+bcpB-1***$^{Chim1}$ | ***+bcpB-1***$^{Chim2}$ | ***+bcpB-1***$^{Chim3}$ | ***+bcpB-1***$^{Chim4}$ | ***+bcpB-1*** | ***+bcpB-2*** |
|---|---|---|---|---|---|---|---|---|
| **Recipient** | ***Δbcp-1*** | ***Δbcp-1*** | ***Δbcp-1*** | ***Δbcp-1*** | ***Δbcp-1*** | ***Δbcp-1*** | ***Δbcp-1*** | ***Δbcp-1*** |
| Exp 1 | 2.13 | -0.72 | -0.12 | 0.29 | -0.28 | 0.39 | -0.36 | 2.83 |
| | 1.75 | -0.31 | -0.43 | 0.13 | -0.13 | 0.60 | -0.14 | 2.89 |
| | 2.83 | -0.60 | 0.68 | 1.04 | -0.66 | 0.61 | -0.18 | 2.95 |
| Exp 2 | 3.88 | 0.45 | 1.19 | 0.48 | -0.68 | 0.80 | -0.45 | 3.88 |
| | 3.70 | -0.01 | 1.38 | 1.08 | -0.82 | 0.54 | -0.50 | 3.41 |
| | 3.95 | -0.32 | 1.21 | 0.23 | -0.52 | 0.66 | -0.44 | 3.48 |
| Exp 3 | 3.56 | 0.31 | 0.88 | 1.27 | 0.05 | 0.48 | -0.24 | 2.46 |
| | 3.62 | -0.22 | 0.92 | 1.13 | -0.09 | 0.41 | -0.19 | 2.55 |
| | 3.42 | -0.38 | 1.11 | 1.41 | -0.17 | 0.29 | -0.17 | 2.58 |

6C

| **Donor** | **WT** | ***Δbcp1-4*** | ***+bcpB-1***$^{Chim1}$ | ***+bcpB-1***$^{Chim2}$ | ***+bcpB-1***$^{Chim3}$ | ***+bcpB-1***$^{Chim4}$ | ***+bcpB-1*** | ***+bcpB-2*** |
|---|---|---|---|---|---|---|---|---|
| **Recipient** | ***Δbcp-2*** | ***Δbcp-2*** | ***Δbcp-2*** | ***Δbcp-2*** | ***Δbcp-2*** | ***Δbcp-2*** | ***Δbcp-2*** | ***Δbcp-2*** |
| Exp 1 | 2.38 | 0.09 | 0.82 | 0.65 | -0.33 | 0.98 | -0.21 | 2.51 |
| | 2.75 | -0.19 | 0.59 | 0.54 | 0.06 | 0.23 | 0.00 | 2.49 |
| | 2.57 | -0.05 | 0.48 | 1.23 | -0.39 | 0.98 | 0.40 | 2.55 |

| | | | | | | | | |
|---|---|---|---|---|---|---|---|---|
| Exp 2 | 2.65 | 0.24 | 1.00 | 0.81 | 0.80 | 0.82 | 0.32 | |
| | 2.15 | 0.29 | 1.00 | 0.43 | 0.42 | 0.70 | 0.03 | |
| | 2.39 | -0.02 | 0.95 | 0.47 | 0.38 | 1.26 | -0.04 | |
| Exp 3 | 2.86 | 0.13 | 1.14 | 0.80 | 0.21 | 0.48 | -0.11 | 2.60 |
| | 2.95 | 0.43 | 0.59 | 1.15 | 0.05 | 0.65 | 0.19 | 2.98 |
| | 2.76 | 0.36 | 1.38 | 0.74 | 0.38 | 0.66 | 0.06 | 2.84 |

7A

| **Donor** | **WT** | ***∆bcpB1-4*** | ***∆bcpB-1*** | ***+bcpB*** $^{E264}$ |
|---|---|---|---|---|
| **Recipient** | ***∆bcp-1*** | ***∆bcp-1*** | ***∆bcp-1*** | ***∆bcp-1*** |
| Exp 1 | 4.07** | 0.52** | 0.42** | 3.38 |
| | 3.78** | 0.38** | 0.35** | 3.90 |
| | 4.02** | 0.91** | 0.37** | 3.54 |
| Exp 2 | 3.90** | -0.38** | 0.44** | 3.33 |
| | 3.48** | -0.33** | 0.37** | 3.61 |
| | 3.78** | -0.47** | -0.06** | 3.67 |
| Exp 3 | 3.85** | -0.25** | 0.04** | 3.37 |
| | 3.70** | -0.34** | 0.04** | 3.22 |
| | 3.78** | -0.37** | 0.26** | 3.05 |

** Values are the same as in 4A (first, second, and third columns) as both experiments (4A and 7A) were performed at the same time using the same controls.

7B

| **Donor** | **WT** | ***∆bcpB1-4*** | ***∆bcpB-1*** | ***+bcpB*** $^{E264}$ |
|---|---|---|---|---|
| **Recipient** | ***∆bcp-1*** | ***∆bcp-1*** | ***∆bcp-1*** | ***∆bcp-1*** |
| Exp 1 | 2.12** | -0.56** | 0.08** | 3.17 |
| | 2.00** | 0.38** | 0.14** | 3.03 |
| | 2.83** | -0.20** | 0.53** | 2.93 |
| Exp 2 | 2.25** | -0.15** | 0.04** | 2.36 |

| | | | | |
|---|---|---|---|---|
| | 2.18** | -0.01** | 0.28** | 2.56 |
| | 2.20** | 0.37** | -0.08** | 2.46 |
| Exp 3 | 2.33** | -0.06** | -0.02** | 1.87 |
| | 1.95** | 0.22** | 0.10** | 2.12 |
| | 1.64** | -0.25** | -0.35** | 2.52 |

** Values are the same as in 4B (first, second, and third columns) as both experiments (4B and 7B) were performed at the same time using the same controls.

8A

| **Donor** | **WT** | ***ΔbcpB1-4*** | ***+bcpB-1$^{Bd}$*** | ***+bcpB-2$^{Bd}$*** | ***+bcpB-3$^{Bd}$*** | ***+bcpB-4$^{Bd}$*** | ***+bcpB-1$^{Bm}$*** | ***+bcpB-2$^{Bm}$*** |
|---|---|---|---|---|---|---|---|---|
| **Recipient** | ***Δbcp-1*** | ***Δbcp-1*** | ***Δbcp-1*** | ***Δbcp-1*** | ***Δbcp-1*** | ***Δbcp-1*** | ***Δbcp-1*** | ***Δbcp-1*** |
| Exp 1 | 3.27 | -0.36 | -0.12 | 3.35 | 1.85 | 3.48 | 0.19 | 3.36 |
| | 3.49 | 0.50 | -0.58 | 3.69 | 2.15 | 3.37 | 0.34 | 3.64 |
| | 3.23 | 0.20 | -0.43 | 3.40 | 2.32 | 3.25 | 0.68 | 3.14 |
| Exp 2 | 3.65 | -0.50 | 0.18 | 3.42 | 2.18 | 3.91 | 0.80 | 3.16 |
| | 3.61 | -0.32 | -0.08 | 3.00 | 1.87 | 3.40 | 0.81 | 3.92 |
| | 3.78 | -0.34 | 0.08 | 3.39 | 2.03 | 3.75 | 0.82 | 3.77 |
| Exp 3 | 3.41 | -0.56 | -0.25 | 3.85 | 0.35 | 3.58 | 1.91 | 3.49 |
| | 3.65 | 0.11 | -0.50 | 3.45 | 0.24 | 3.44 | 1.75 | 3.49 |
| | 3.80 | -0.13 | -0.08 | 3.45 | 0.67 | 4.01 | 1.82 | 3.30 |

8B

| **Donor** | **WT** | ***ΔbcpB1-4*** | ***+bcpB-1$^{Bd}$*** | ***+bcpB-2$^{Bd}$*** | ***+bcpB-3$^{Bd}$*** | ***+bcpB-4$^{Bd}$*** | ***+bcpB-1$^{Bm}$*** | ***+bcpB-2$^{Bm}$*** |
|---|---|---|---|---|---|---|---|---|
| **Recipient** | ***Δbcp-2*** | ***Δbcp-2*** | ***Δbcp-2*** | ***Δbcp-2*** | ***Δbcp-2*** | ***Δbcp-2*** | ***Δbcp-2*** | ***Δbcp-2*** |
| Exp 1 | 2.75 | -0.01 | -0.14 | 2.44 | 2.27 | 2.77 | 0.37 | 2.04 |
| | 2.65 | -0.06 | -0.39 | 2.54 | 1.74 | 2.48 | 0.52 | 2.32 |
| | 2.27 | -0.07 | -0.37 | 2.63 | 2.19 | 2.47 | 0.36 | 2.23 |
| Exp 2 | 2.52 | 0.62 | 0.48 | 2.54 | 3.21 | 2.92 | 0.77 | 3.25 |
| | 2.38 | 0.60 | 0.05 | 2.80 | 2.93 | 2.93 | 0.99 | 3.39 |
| | 2.37 | 0.70 | 0.38 | 2.66 | 2.82 | 2.81 | 0.83 | 3.30 |

| | | | | | | | | |
|---|---|---|---|---|---|---|---|---|
| Exp 3 | 1.63 | 0.24 | -0.08 | 2.14 | 2.50 | 2.65 | 0.75 | 3.18 |
| | 1.62 | 0.02 | -0.23 | 2.26 | 2.47 | 2.85 | 0.59 | 2.99 |
| | 1.94 | -0.44 | -0.59 | 2.09 | 2.71 | 3.08 | 0.33 | 2.87 |

8C

| **Donor** | **WT** | ***Δbcp-1*** | ***ΔbcpB-2*** | ***Δbcp-1 ΔbcpB-2*** | ***+bcpB-1*** $^{Bm}$ | ***+bcpB-2*** $^{Bm}$ |
|---|---|---|---|---|---|---|
| **Recipient** | ***Δbcp-1 Δbcp-2*** | ***Δbcp-1 Δbcp-2*** | ***Δbcp-1 Δbcp-2*** | ***Δbcp-1 Δbcp-2*** | ***Δbcp-1 Δbcp-2*** | ***Δbcp-1 Δbcp-2*** |
| Exp 1 | 1.92 | 1.76 | 0.97 | 0.23 | 0.72 | 2.12 |
| | 1.86 | 1.9 | 0.82 | -0.19 | -0.37 | 1.21 |
| | 2.00 | 1.88 | 0.69 | 0.15 | 0.98 | 2.21 |
| Exp 2 | 2.1 | 1.97 | 0.73 | 0.09 | 1.02 | 1.99 |
| | 2.05 | 2.48 | 0.66 | 0.06 | 1.49 | 2.05 |
| | 2.36 | 2.41 | 0.95 | -0.17 | 1.65 | 2.05 |
| Exp 3 | 1.12* | 1.21* | 0.18* | -0.10* | 0.89 | 1.43 |
| | 1.19* | 1.06* | 0.12* | 0.10* | 0.65 | 1.07 |
| | 1.40* | 1.12* | -0.37* | -0.10* | 0.85 | 1.67 |

* Values are the same as in 8D Exp.1 (columns 1-4) as both experiments (8C Exp. 3 and 8D Exp.1) were performed at the same time using the same controls.

8D

| **Donor** | **WT** | ***Δbcp-1*** | ***ΔbcpB-2*** | ***Δbcp-1 ΔbcpB-2*** | ***+bcpB-1*** $^{Bd}$ | ***+bcpB-2*** $^{Bd}$ | ***+bcpB-3*** $^{Bd}$ | ***+bcpB-4*** $^{Bd}$ |
|---|---|---|---|---|---|---|---|---|
| **Recipient** | ***Δbcp-1 Δbcp-2*** | ***Δbcp-1 Δbcp-2*** | ***Δbcp-1 Δbcp-2*** | ***Δbcp-1 Δbcp-2*** | ***Δbcp-1 Δbcp-2*** | ***Δbcp-1 Δbcp-2*** | ***Δbcp-1 Δbcp-2*** | ***Δbcp-1 Δbcp-2*** |
| Exp 1 | 1.12* | 1.21* | 0.18* | -0.10* | 0.11 | 0.79 | 1.01 | 1.71 |
| | 1.19* | 1.06* | 0.12* | 0.10* | -0.11 | 0.58 | 1.24 | 1.96 |
| | 1.40* | 1.12* | -0.37* | -0.10* | 0.92 | 0.69 | 1.24 | 1.59 |
| Exp 2 | 1.28 | 1.95 | 0.21 | -0.17 | 0.24 | 0.48 | 1.7 | 2.31 |
| | 1.21 | 1.28 | 0.83 | -0.46 | -0.02 | 0.78 | 1.68 | 1.91 |
| | 1.36 | 1.33 | 0.42 | 0.05 | 0.3 | 0.48 | 1.88 | 1.9 |

| | | | | | | | | |
|---|---|---|---|---|---|---|---|---|
| Exp 3 | 1.96 | 2.11 | 0.87 | 0.07 | 0.3 | | 1.89 | 2.1 |
| | 2.36 | 3.23 | 0.9 | 0.23 | -0.03 | 0.58 | 2.13 | 1.88 |
| | 2.34 | 2.08 | 0.59 | 0.11 | | 0.67 | 2.12 | 1.99 |

* Values are the same as in 8C Exp.3 (columns 1-4) as both experiments (8C Exp. 3 and 8D Exp.1) were performed at the same time using the same controls.

**Supplemental References**


1. Anderson MS, Garcia EC, Cotter PA. 2012. The *Burkholderia bcpAIOB* genes define unique classes of two-partner secretion and contact dependent growth inhibition systems. PLoS genetics 8:e1002877-e1002877. 10.1371/journal.pgen.1002877.
2. Choi K-H, Mima T, Casart Y, Rholl D, Kumar A, Beacham IR, Schweizer HP. 2008. Genetic tools for select-agent-compliant manipulation of *Burkholderia pseudomallei*. Applied and Environmental Microbiology 74:1064-1075. 10.1128/AEM.02430-07.
3. Elery ZK, Oates AE, Myers-Morales T, Garcia EC. 2022. Recipient cell factors influence interbacterial competition mediated by two distinct *Burkholderia dolosa* contact-dependent growth inhibition systems. Journal of Bacteriology 204 10.1128/jb.00541-21.
4. Perault AI, Cotter PA. 2018. Three distinct contact-dependent growth inhibition systems mediate interbacterial competition by the cystic fibrosis pathogen *Burkholderia dolosa*. Journal of bacteriology 200 https://doi.org/10.1128/jb.00428-18.
5. Myers-Morales T, Oates AE, Byrd MS, Garcia EC. 2019. *Burkholderia cepacia* complex contact-dependent growth inhibition systems mediate interbacterial competition. Journal of bacteriology 201 10.1128/JB.00012-19.
6. López CM, Rholl DA, Trunck LA, Schweizer HP. 2009. Versatile dual-technology system for markerless allele replacement in *Burkholderia pseudomallei*. Applied and environmental microbiology 75:6496-503. 10.1128/AEM.01669-09.
7. Choi K-H, Gaynor JB, White KG, Lopez C, Bosio CM, Karkhoff-Schweizer RR, Schweizer HP. 2005. A Tn7-based broad-range bacterial cloning and expression system. Nature methods 2:443-8. 10.1038/nmeth765.
